# Characterization of Properties and Transglycosylation Abilities of Recombinant α-Galactosidase from Cold-Adapted Marine Bacterium *Pseudoalteromonas* KMM 701 and Its C494N and D451A Mutants

**DOI:** 10.3390/md16100349

**Published:** 2018-09-24

**Authors:** Irina Bakunina, Lubov Slepchenko, Stanislav Anastyuk, Vladimir Isakov, Galina Likhatskaya, Natalya Kim, Liudmila Tekutyeva, Oksana Son, Larissa Balabanova

**Affiliations:** 1 Laboratory of Enzyme Chemistry, Laboratory of Marine Biochemistry, Laboratory of Bioassays and Mechanism of action of Biologically Active Substances, Laboratory of Instrumental and Radioisotope Testing Methods, Group of NMR-Spectroscopy of G.B. Elyakov Pacific Institute of Bioorganic Chemistry, Far Eastern Branch, Russian Academy of Sciences, Vladivostok 690022, Russia; lubov99d@mail.ru (L.S.); Sanastyuk@piboc.dvo.ru (S.A.); ivv43@mail.ru (V.I.); galin56@mail.ru (G.L.); natalya_kim@mail.ru (N.K.); lbalabanova@mail.ru (L.B.); 2 School of Economics and Management, School of Natural Sciences of Far Eastern Federal University, Russky Island, Vladivostok 690022, Russia; tekuteva.la@dvfu.ru (L.T.); oksana_son@bk.ru (O.S.)

**Keywords:** α-d-galactosidase, homology model, GH 36 family, mutation, transglycosylation, marine bacteria, *Pseudoalteromonas* sp. KMM 701

## Abstract

A novel wild-type recombinant cold-active α-d-galactosidase (α-PsGal) from the cold-adapted marine bacterium *Pseudoalteromonas* sp. KMM 701, and its mutants D451A and C494N, were studied in terms of their structural, physicochemical, and catalytic properties. Homology models of the three-dimensional α-PsGal structure, its active center, and complexes with D-galactose were constructed for identification of functionally important amino acid residues in the active site of the enzyme, using the crystal structure of the α-galactosidase from *Lactobacillus acidophilus* as a template. The circular dichroism spectra of the wild α-PsGal and mutant C494N were approximately identical. The C494N mutation decreased the efficiency of retaining the affinity of the enzyme to standard p-nitrophenyl-α-galactopiranoside (pNP-α-Gal). Thin-layer chromatography, matrix-assisted laser desorption/ionization mass spectrometry, and nuclear magnetic resonance spectroscopy methods were used to identify transglycosylation products in reaction mixtures. α-PsGal possessed a narrow acceptor specificity. Fructose, xylose, fucose, and glucose were inactive as acceptors in the transglycosylation reaction. α-PsGal synthesized -α(1→6)- and -α(1→4)-linked galactobiosides from melibiose as well as -α(1→6)- and -α(1→3)-linked p-nitrophenyl-digalactosides (Gal_2_-pNP) from pNP-α-Gal. The D451A mutation in the active center completely inactivated the enzyme. However, the substitution of C494N discontinued the Gal-α(1→3)-Gal-pNP synthesis and increased the Gal-α(1→4)-Gal yield compared to Gal-α(1→6)-Gal-pNP.

## 1. Introduction

α-d-Galactosidases (EC 3.2.1.22) catalyze the hydrolysis of the nonreducing terminal α-d-galactose (Gal) from α-d-galactosides, galactooligosaccharides, and polysaccharides, such as galactomannans, galactolipids, and glycoproteins. According to the classification of carbohydrate-active enzymes (CAZy) [1], α-d-galactosidases mostly belong to 27, 36, and 110 families of glycoside hydrolases (GH). They are found also in the GH 4, GH 57, and GH 97 families. The GH 27 and GH 36 enzymes, with a common mechanism of catalysis, have the protein structural (β/α)_8_-barrel fold in the catalytic domain and similar topology of their active centers, typical for a clan GH D [2]. The GH 27 and GH 36 family members are classical retaining glycoside hydrolyses in accordance with Koshland’s classification [3]. These enzymes catalyze the hydrolysis of O-glycosidic bonds by a double displacement mechanism through the galactosyl-enzyme covalent intermediate, as well as the transglycosylation reaction under the specific conditions [4].

α-d-Galactosidases are widespread among terrestrial plants, animals, and microorganisms. These enzymes have found many practical uses in different fields from biomedicine to enzymatic synthesis [4]. The enzymes occur frequently in marine bacteria, especially in γ-Proteobacteria and Bacteroidetes [5,6,7,8,9]. Currently, the genes encoding these enzymes can be found in the genomes of marine bacteria, annotated in the National Center for Biotechnology Information NCBI database. For the first time, α-PsGal was isolated from a cold-adapted marine bacterium *Pseudoalteromonas* sp. strain KMM 701 inhabiting in the cold water in the Sea of Okhotsk. The enzyme attracted our attention due to its ability to reduce the serological activity of B red blood cells. The marine bacterium’s α-PsGal was more efficient in the model of B-erythrocyte antigen, than a well-known α-d-galactosidase from green coffee beans, which was usually used in experiments on transformation of donor blood erythrocytes for intravenous injection [10]. The enzyme also interrupted the adhesion of *Corynebacterium diphtheria* to buccal epithelium cells at neutral pH values [11], as well as stimulated the growth of biofilms of some bacteria [12]. These properties of the enzyme determined the possible directions for its practical application in biomedicine. According to the structural CAZy classification, α-PsGal belongs to the GH 36 family [11]. The enzyme is a retaining glycoside hydrolase [13], cleaving the terminal Gal from melibiose Gal-α(1→6)-Glc, raffinose Gal-α(1→6)-Glc-β(1→4)-(Fru), digalactoside Gal-α(1→3)-Gal, and B-trisaccharide Gal-α(1→3)-(Fuc-α(1→2)-Gal) [10]. However, the most important glycosynthase properties of the enzyme have yet to be studied.

The present article aimed to compare the properties of recombinant α-d-galactosidase from marine bacterium *Pseudoalteromonas* sp. KMM 701 (α-PsGal) and its mutants, where the predicted functionally important residues D451 and C494 of the active center were replaced by the less reactive alanine (A) and asparagine (N) residues, respectively. Major attention was focused on the regioselectivity of the transglycosylation reaction.

## 2. Results

### 2.1. Bioinformatics Analysis and Homology Modeling of α-PsGal Protein 3D-Structure

**Figure 1:**
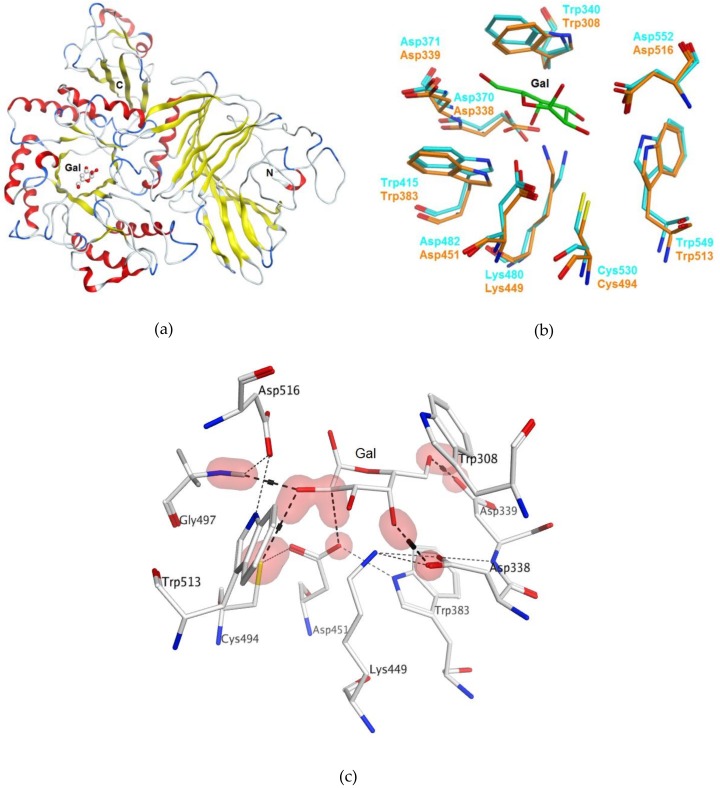
Homology model of α-PsGal three-dimensional (3D) structure generated using X-ray structure of the α-galactosidase of *Lactobacillus acidophilus* (PDB ID 2XN2) as a template: (**a**) 3D-model of α-PsGal structure in a ribbon diagram representation: α-helixes (red), β-strands (yellow), coils (white), and turns (blue); (**b**) superimposition of the α-PsGal homology model (orange) with template active sites (turquoise); D-galactose is shown by sticks (green); and (**c**) the binding site of D-galactose in the active center of α-PsGal homology model. Hydrogen-bond contacts were determined using the Protein Contacts module of Molecular Operating Environment version 2018.01 (MOE) program (Chemical Computing Group ULC: 1010 Sherbrooke St. West, Suite #910, Montreal, QC, Canada, H3A 2R7, 2018) and are shown with a dashed line.

Bioinformatics analysis and homology modeling of the protein structure was completed to elucidate the amino acid residues roles of α-PsGal for catalysis. The results of homology modeling of the α-PsGal protein three-dimensional (3D) structure are shown in Figure 1.

The homology model of the α-PsGal 3D-structure was constructed by the package Molecular Operating Environment version 2018.01 (MOE) [14] using the crystal structure of α-galactosidase from *Lactobacillus acidophilus* of the GH 36 family [15] as a template (Figure 1a). The amino acid sequence of α-galactosidase from the marine bacterium has 28.7% identity and 44% similarity with the sequence of the prototype. The superposition (root mean squared difference (RMSD) of the C_α_-atoms = 0.8 Å) of the α-PsGal homology model with the template active site revealed that the D451 and C494 residues superimposed on the nucleophile/base D482 and the substrate binding C530 residues in the template, respectively (Figure 1b) [15]. Thus, the predicted structure of α-PsGal is applicable for in silico mutagenesis and molecular docking studies. The evidence from Figure 1c suggests that C494 takes part in forming a network of hydrogen bonds between the catalytic residues D451, D516, and the hydrolysis product D-galactose (D-Gal). To eliminate the roles of D451 and C494 residues, they were substituted by A451 and N494, respectively.

### 2.2. Enzyme Production and Purification

**Figure 2:**
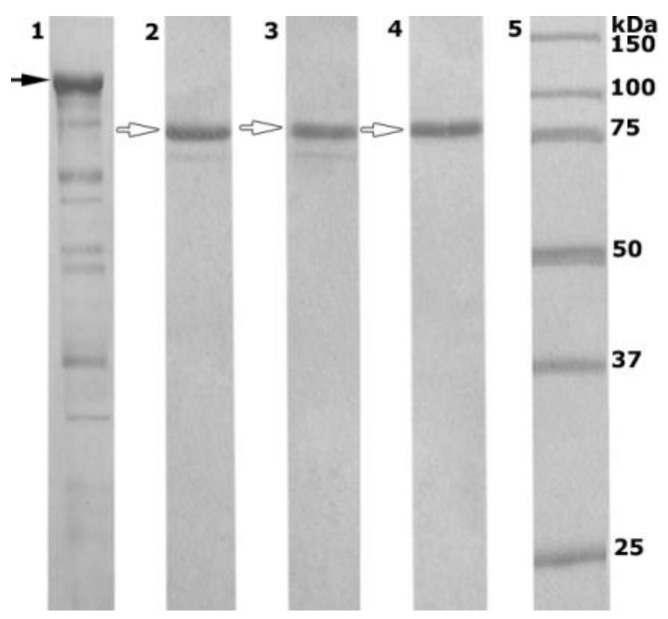
SDS-PAGE (12.5%) of the recombinant wild α-PsGal (lanes 1 and 2 before and after final purification stage, respectively) and its D451A and C494N mutants after the final stages of purification (lanes 3 and 4, respectively); molecular weight markers are shown in lane 5.

The recombinant wild α-PsGal and its mutants D451A and C494N were expressed and purified successfully as soluble proteins with 97% purity according to the sodium dodecyl sulfate polyacrylamide gel electrophoresis (SDS-PAGE) data. The gel electorophoregrams of the enzyme preparations were obtained in different experiments and are summarized in Figure 2.

The molecular weight of the protein fraction of the cellular extract *Escherichia coli* Rosetta (DE3)/40Gal (Figure 2, lane 1) corresponds to the chimeric recombinant α-PsGal fused with the plasmid pET-40 b (+) chaperone protein DsbC overhang (80 kDa (α-PsGal) + 32.5 kDa (DsbC) = 112.5 kDa). After the final stage of purification and treatment with enterokinase, the molecular weight of mature recombinant proteins α-PsGal, D451A, and C494N were ca. 80 kDa (Figure 2, lanes 2–4, respectively). 

### 2.3. Properties of Recombinant Wild-Type α-PsGal and Mutant C494N

The mutant D451A did not exhibit any hydrolytic activity against either melibiose or pNP-α-Gal, indicating the extreme importance of this residue for the functioning of this enzyme. The specific activities of the recombinant wild α-PsGal and mutant C494N, with the use of pNP-α-Gal as a substrate, were 90.0 and 0.87 U/mg, respectively. Further comparative studies showed some similarities and differences in the enzymatic properties of the wild recombinant α-PsGal and its mutant C494N.

#### 2.3.1. Circular Dichroism Spectra of Wild-Type α-PsGal and Mutant C494N

**Figure 3:**
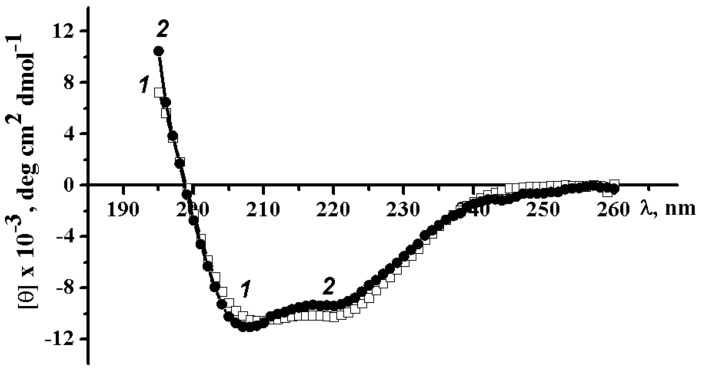
Circular dichroism spectra of wild α-PsGal (1) and C494N mutant (2) with 0.1 M sodium phosphate buffer (pH 7.0), 25 °C, and 0.1 cm cell.

To identify the similarity of the secondary structure of the wild α-PsGal and C494N mutant; circular dichroism (CD) spectroscopy was used (Figure 3).

The CD spectra of the wild α-PsGal and C494N mutant were approximately identical binshape and amplitude of the bands (Figure 3, spectrum 1 and 2, respectively). Calculation of the secondary protein structure elements (see p. 4.4.1.) indicated the presence of 27.9% and 27.9% α-helices, 19.9% and 21.4% β-structures, and 28.9% and 28.9% disordered structure, including 23.3% and 21.8% β-turns for the wild α-PsGal and C494N mutant, respectively. The determination of the tertiary structure class using the same software package established that the wild α-PsGal and C494N mutant belong to α + β tertiary structure class of proteins. Thus, the C494N point mutation in the active site does not significantly affect the secondary structure of the enzyme as a whole.

#### 2.3.2. Effect of pH on Wild-Type α-PsGal and C494N Mutant Activities

**Figure 4:**
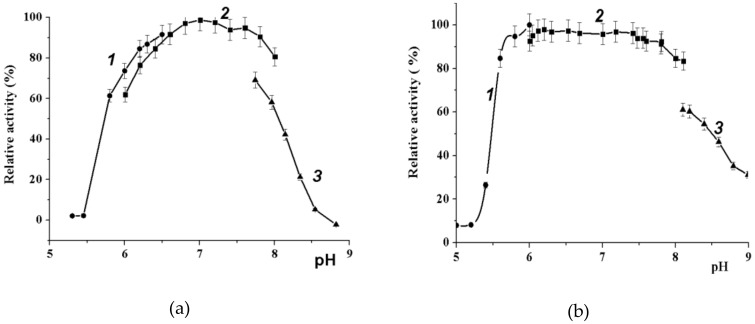
Effect of pH on the activity of enzymes: (**a**) wild-type α-PsGal and (**b**) mutant C494N. Fragments of curves correspond to 0.1 M sodium citrate buffer (1), 0.1 M sodium phosphate buffer (2), and 0.1 M Tris HCl buffer (3).

It is evident from Figure 4 that the wild α-PsGal (Figure 4a) retains activity in a wide pH range (6.5–8.0).

Citrate and phosphate anions were preferable for enzyme activity. The tris ion was an effective inhibitor of the activity of wild α-PsGal and mutant C494N. The replacement of cysteine 494 with asparagine residue did not significantly change the pH effect on the enzyme activity (Figure 4).

#### 2.3.3. Effect of Temperature on Wild-Type α-PsGal and Mutant C494N Activities

**Figure 5:**
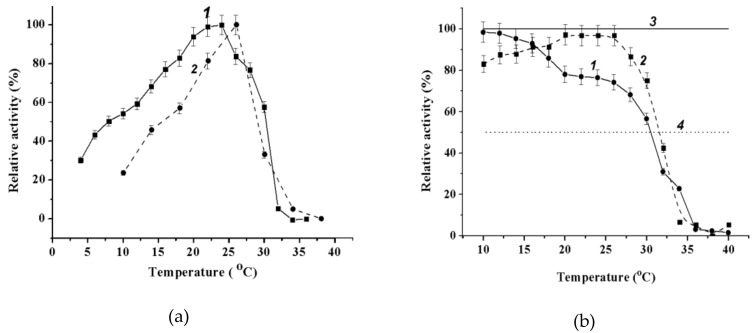
Effect of temperature on activity of enzymes: (**a**) the dependence of relative activity on temperature of wild α-PsGal (1) and C494N mutant (2) and (**b**) thermal stability of wild α-PsGal (1) and C494N mutant (2). The solid line (3) indicates 100% activity and the dashed line (4) indicates 50% activity.

It is evident that wild α-PsGal is a cold-active enzyme due to retaining about 30% of its activity at 5 °C (Figure 5a). The activity of the mutant C494N reached a maximum at higher temperature values than wild α-PsGal (Figure 5b). 

Although the middle of the temperature transition lay at the same temperature of ~32 °C, the temperature inactivation in the wild α-PsGal started at lower temperatures than in the C494N mutant. Both the recombinant wild α-PsGal and the C494N mutant proved to be more thermostable enzymes than the natural α-galactosidase from the marine bacterium *Pseudoalteromonas* sp. KMM 701 [10].

#### 2.3.4. Kinetic Parameters of Catalytic Reactions for Wild-Type α-PsGal and Mutant C494N

Michaelis-Menten constant (*K*_m_) and maximal rate (*V*_max_) of pNP-α-Gal hydrolysis were defined from Lineweaver-Burk graphs.

Catalytic parameters of the reaction are summarized in Table 1.

It is evident from Table 1 that the replacement of cysteine 494 in the active site of α-PsGal with asparagine residue leads to an approximately 200-fold decrease in the efficiency (*k*_cat_/*K*_m_) of the enzyme retaining the identical affinity (*K*_m_) of both enzymes to the standard substrate pNP-α-Gal. This indicates the extreme importance of C494 in the manifestation of α-PsGal activity (Table 1).

#### 2.3.5. Theoretical Model of the D-Gal Complexes with Wild α-PsGal and Mutant C494N

**Figure 6:**
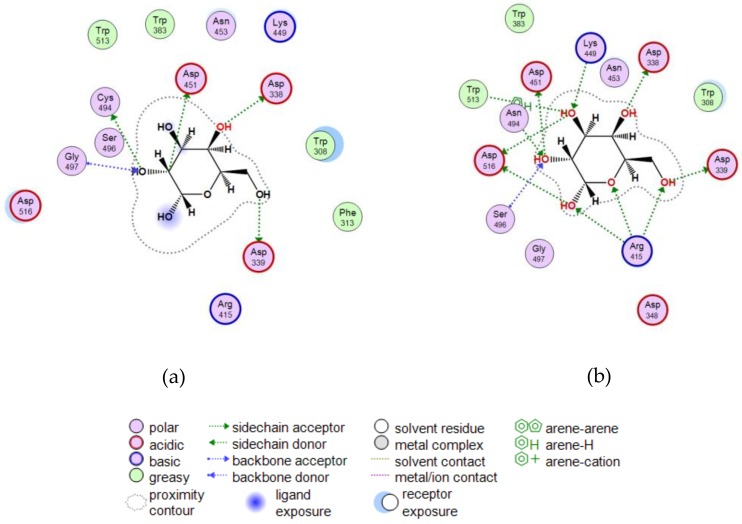
Two-dimensional (2D) diagrams of the D-Gal binding sites in (**a**) wild α-PsGal and (**b**) mutant C494N.

Figure 6 shows two-dimensional (2D) diagrams of the D-Gal complexes with the active center of the wild α-PsGal (Figure 6a) and mutant C494N (Figure 6b) built by molecular docking in the MOE program.

In silico analysis of the wild α-PsGal-d-Gal and mutant C494N-d-Gal complexes (Figure 6) showed that substitution C494N led to the emergence of new hydrogen bonds (Figure 6b) and to the increase in binding energy of the reaction product D-Gal in the binding site of the enzyme (Table S1). This probably reflected a decrease in the values of the *k*_cat_ and *k*_cat_/*K*_m_ constants of the enzyme.

### 2.4. Acceptor Specificity of Recombinant α-PsGal

The results of the preliminary determination of the acceptor specificity in the transglycosylation reaction of the wild α-PsGal showed that the enzyme possesses narrow acceptor specificity, as new sugars fructose, xylose, fucose, and glucose were not found in the experimental conditions (Table 2).

### 2.5. Production of Transglysolylation Reactions

The transglycosylation properties of the purified wild α-PsGal, mutant D451A, and C494N were tested using melibiose (1.10 mmol/mL) and pNP-α-Gal (0.25 mmol/mL) as substrates at pH 7.0 and 8 °C and 20 °C. The concentration of the substrates significantly exceeded the enzyme concentration. The composition of incubation mixtures and units of the enzymes are shown in Table 3. 

Since the activity of mutant C494N was much lower compared with the wild type (Table 3), the reaction mixtures containing weakly active C494N (0.012 U) and wild-type α-PsGal with an activity of 0.015 U were incubated for seven days. In order to avoid the thermal inactivation of the enzymes, the reactions were carried out in a refrigerator at 8 °C. Therefore, the products from the reactions at low (8 °C) and moderate (20 °C) temperatures were compared. The conditions of rescue experiments and the investigation of glycosynthase properties of the mutant D451A in the presence of sodium azide, as an external nucleophile, are also shown in Table 3. 

#### 2.5.1. Thin Layer Chromatography (TLC)

**Figure 7:**
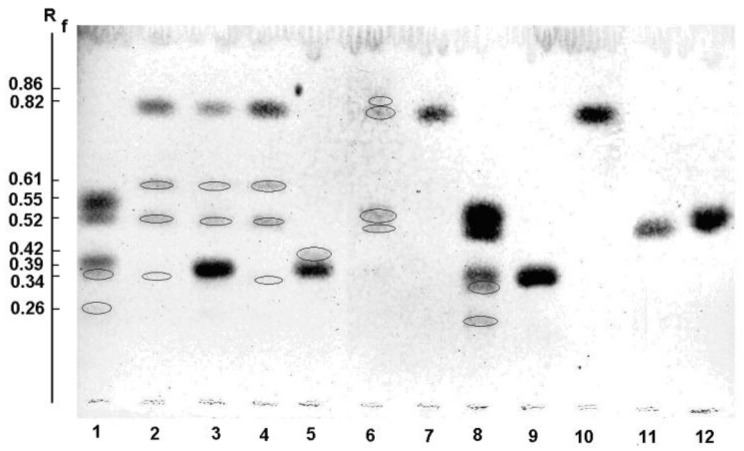
Thin layer chromatography (TLC) profiles of the hydrolysis and transglycosylation products produced by recombinant α-PsGal. Lanes 1 and 8—α-PsGal with Gal-α(1→6)-Glcα,β at 20 °C and 8 °C, respectively. Lanes 2 and 4—α-PsGal with pNP-α-Gal at 20 °C and 8 °C, respectively. Lane 3—α-PsGal with mixture of Gal-α(1→6)-Glcα,β/pNP-α-Gal at 20 °C. Lane 5—D451A mutant with Gal-α(1→6)-Glcα,β/NaN_3_. Lane 6—D451A mutant with pNP-α-Gal/NaN_3_. Lane 7—D451A mutant with pNP-α-Gal. Blank mixtures: lane 9—Gal-α(1→6)-Glcα,β, lane 10—pNP-α-Gal, lane 11—Gal, and lane 12—Glc.

The results of TLC analyses of the reaction mixtures after the action of recombinant α-PsGal, as well as D451A and C494N mutants, on melibiose (Gal-α(1→6)-Glc), and pNP-α-Gal were obtained under different conditions (Figure 7).

Lanes 1–8 correspond to each experiment. The melibiose, pNP-α-Gal, galactose (Gal), and glucose (Glc) standards (Figure 7, lanes 9–12, respectively) spots on the chromatogram had retardation factors (R_f_) of 0.39, 0.83, 0.52, and 0.56, respectively. The conditions of reactions are listed in Table 3.

After separation of the reaction mixtures with the melibiose processed by the recombinant α-PsGal at 20 °C and 8 °C (Figure 7; lanes 1 and 8; respectively), two new spots with R_f_ 0.35 and 0.26 occurred in the chromatogram; in addition to the hydrolysis products Gal and Glc (R_f_ 0.52 and 0.55; respectively). These new sugars could be interpreted as transglycosylation products. In the mixture of the reaction products of pNP-α-Gal treated with the recombinant α-PsGal at 20 °C and 8 °C; the spots with R_f_ 0.61 and 0.34, corresponding to new sugars along with the spots of pNP-α-Gal and Gal (R_f_ = 0.82 and 0.52; respectively), were observed (Figure 7; lanes 2 and 4; respectively). It is interesting to note that the compound with the R_f_ value of 0.34 was not formed by the action of the recombinant wild α-PsGal on the mixture of melibiose (donor)/pNP-α-Gal (acceptor) (Figure 7; lane 3). The D451A mutant showed no activity toward pNP-α-Gal (Figure 7; lane 7). Unfortunately, reactivation experiments with external nucleophile sodium azide failed to restore the activity of the nucleophile mutant enzyme (data not shown). However, in the presence of sodium azide (Figure 7; lanes 5 and 6), the traces of unidentified compounds (R_f_ value of 0.42 and 0.86; respectively) were formed under an action of D451A on melibiose and pNP-α-Gal

#### 2.5.2. MALDI Mass Spectrometry

Matrix-assisted laser desorption/ionization (MALDI) mass spectra were recorded for five samples (S1): α-PsGal with a mixture of melibiose and pNP-α-Gal, α-PsGal with pNP-α-Gal, D451A with pNP-α-Gal and NaN_3_, C494N with pNP-α-Gal, as well as standard mixtures: melibiose, pNP-α-Gal, Gal, and Glc. The molecular weights of sugars (both products of hydrolysis and substrates) were registered as sodium adducts [M + Na]^+^ in positive-ion mode, where M represents the neutral molecule: [Hex + Na]^+^ at *m*/*z* 203.06, [Hex_2_ + Na]^+^ at *m*/*z* 365, [pNP-Hex_2_ + Na]^+^ at *m*/*z* 486, and [Hex_3_ + Na]^+^ at *m*/*z* 527. pNP-α-Gal was found as [pNP-α-Gal + Na]^+^ ion at *m*/*z* 324. MALDI mass spectra showed only the semiqualitative composition of the transglycosylation products (Table 4).

According to the mass spectral data, the signal of the [Hex_2_ + Na]^+^ ion at *m*/*z* 365 of disaccharide (Hex_2_) was major in the MALDI MS of the reaction mixture of α-PsGal with melibiose/pNP-α-Gal. In addition, there were signals of the new [pNP-Hex_2_ + Na]^+^ and [Hex + Na]^+^ ions in the spectrum (Figure S1a) at *m*/*z* 486 and 203, respectively. Along with signals of the hydrolysis product [Hex + Na]^+^ ion at *m*/*z* 203 and the remainder of the substrate [pNP-Hex + Na]^+^ ion at *m*/*z* 324, new signals of [Hex_2_ + Na]^+^ and [pNP-Hex_2_ + Na]^+^ ions were observed in the matrix-assisted laser desorption ionization-mass spectroscopy (MALDI-MS) of the reaction mixture of the wild α-PsGal with pNP-α-Gal at 8 °C (Figure S1b). The signals of new carbon compounds were not detected in the MALDI-MS of the product mixture obtained under the action of mutant D451A on pNP-α-Gal in the presence of NaN_3_ (Figure S1c). The main signal of the [Hex_2_ + Na]^+^ ion at *m*/*z* 365 was identified in the spectrum of the reaction mixture of the C494N mutant with pNP-α-Gal. The spectrum also contained two minor signals of [pNP-Hex_2_ + Na]^+^ at *m*/*z* 486 and [Hex + Na]^+^ ion at *m*/*z* 203 (Figure S1d).

To identify the regioselectivity of the transglycosylation, we used tandem electrospray ionization mass spectrometry (EISMS/MS) with collisional induced dissociation (CID) in positive ion mode. The EISMS profiles of the reaction products are illustrated in Figures S2, S3, and S4. The linkage identifications were based on the fragmentation rules described earlier [16] for negative ion mode, and further supported by positive ion mode [17]. In brief, the absence of fragment ions from cross-ring cleavages in a disaccharide suggests a 1,3-type linkage, the ^0,2^A_2_-type fragment ion suggests 1,4-type linkages, and both ^0,2^A_2_ and ^0,3^A_2_-type ions suggest 1,6-type linkages in disaccharides. The nomenclature for the mass spectrometric fragmentation of glycoconjugates was suggested by Domon and Costello [18].

Ion signals of [Hex_n_ + Na]^+^, *n* = 1–3, at *m*/*z* 203, 365, and 527 were the major components found by EISMS among the reaction products of melibiose and α-PsGal (Figure S2a). The CID ESIMS/MS fragmentation pattern of a trisaccharide ion suggested Hex-(1→4)-Hex-(1→6)-Hex structure (Figure S2b). Fragment ion ^0,2^A_2_ at *m*/*z* 305 and ^0,3^A_2_ at *m*/*z* 275 indicated the presence of both 1→4- and 1→6-*O*-glycosidic links between two hexoses in disaccharide (Figure S2c). The question concerning the presence of the (1→3)-linked hexoses remained unclear, since the disaccharide Galα-(1→3)-Gal could be identified only by the absence of fragment ions from cross-ring cleavages [16]. Ion signals of [Hex_n_ + Na]^+^, *n* = 1,2, at *m*/*z* 203 and 365 were the major components found by EISMS among the reaction products of melibiose and mutant C494N (Figure S3a). The CID ESIMS/MS fragmentation pattern of a disaccharide ion suggested Hex-(1→4)-Hex only (Figure S3b), so the fragment ion ^0,2^A_2_ at *m*/*z* 305 was observed. The type of *O*-glycosidic bond in the pNP-glycosides could not be established because the mobile proton at the glycosyl hydroxyl was blocked. In this case, fragmentation was not observed (Figure S3c–e and Figure S4b) [19].

**Figure 8:**
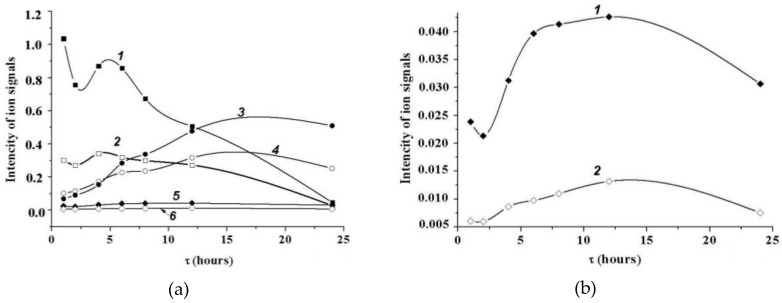
Mass spectrometry monitoring of the reaction of melibiose hydrolysis and transglycosylation catalyzed by wild α-*Ps*Gal in the buffered heavy-oxygen water at 20 °C. (**a**) Experimental time-dependent changes in integral intensity of matrix-assisted laser desorption ionization-mass spectroscopy (MALDI-MS) signals of the Hex_2_ ions at 365 *m*/*z* (1) and 367 *m*/*z* (2), Hex at 203 *m*/*z* (3) and 205 *m*/*z* (4), and Hex_3_ at 527 *m*/*z* (5) and 529 *m*/*z*; (**b**) time dependences of the MALDI-MS signals intensity of Hex_3_ ions at 527 *m*/*z* (1) and 529 *m*/*z* (2) in an expanded scale.

We used heavy ^18^O-water for the transglycosylation experiment; we deemed it the most interesting substrate. The use of buffered heavy-oxygen water and liquid chromatography (LC), coupled with ESI-MS/MS (LC-ESIMS/MS) allowed simultaneous observing of the products of hydrolysis and transglycosylation. Since the transfer of heavy ^18^OH-group produces a +2 mass shift, it was possible to distinguish between fragment ions, retaining the positive charge on the reducing end from the charge on the nonreducing end. Figure 8 shows the kinetics of the consumption and accumulation of the hydrolysis and synthesis products with the use of heavy-oxygen water.

According to the results (Figure 8), the consumption of Hex_2_ (Figure 8a, curves 1 and 2) was accompanied by the appearance of Hex (Figure 8a, curves 3 and 4), new Hex_2_ (Figure 8a, curves 1 and 2), and Hex_3_ (Figure 8b).

#### 2.5.3. NMR Spectroscopy

Nuclear magnetic resonance (NMR) spectroscopy was used to elucidate the transglycosylation regioselectivity and product structures. To avoid loss of information about minor transglycosylation products, the proton (^1^H) and carbon (^13^C) NMR spectra were analyzed without separating the reaction mixtures into individual compounds. The ^1^H and ^13^C signals of the anomeric atoms of the substrates and the products were assigned according to the respective reference data [20,21,22], as well as by H,H-Correlation Spectroscopy (COSY) and Heteronuclear Single-Quantum Correlation (HSQC) experiments. The identified signals are shown in Table 5.

The composition and yields of transglycosylation products in each case are shown in Table 6.

In the ^1^H NMR spectrum of the reaction mixture obtained after the action of recombinant α-PsGal on melibiose, the α,β1H signals of galactose (Gal) (**1**) and glucose (Glc) (**2**), which are the hydrolysis products, along with the major α,β1H signal of transglycosylation product Gal-α(1→6)-Galα,β (**3**) and a minor signal of Gal-α(1→4)-Galα,β (**4**), were observed (Table 5 and Table 6). In the ^1^H NMR spectrum of the products obtained after the action of recombinant α-PsGal on pNP-α-Gal, the signals of the α1H atoms were registered and characterized for the major autocondensation product Gal-α(1→6)-Gal-α-pNP (**6**) and a minor autocondensation product Gal-α(1→3)-Gal-α-pNP (**7**), as well as for the transglycosylation products Gal-α(1→6)-Galα,β (**3**) and Gal-α(1→4)-Galα,β (**4**) (Table 5 and Table 6). The substituted bigalactoside Gal-α(1→6)-Gal-α-pNP (**6**) was a major product of transglycosylation obtained under the action of the recombinant α-PsGal on the mixture of melibiose (donor) and pNP-α-Gal (acceptor), whereas unsubstituted bigalactosides, Gal-α(1→6)-Galα,β (**3**) and Gal-α(1→4)-Galα,β (**4**), were synthesized in small amounts. Signals of α1H of Gal-α(1→6)-Gal-α-pNP (**6**) were found in the ^1^H NMR spectrum of reaction products observed in the reaction mixture with the C494N mutant with pNP-α-Gal as the substrate. In the last case, there were no α1H signals found for compound Gal-α(1→3)-Gal-α-pNP (**7**). Analysis of the reaction mixture after the action of the D451A mutant on pNP-α-Gal did not reveal new signals in the NMR spectra except for the signals corresponding to pNP-α-Gal.

## 3. Discussion

The catalytic properties and structure-function relationships for the marine bacterial α-galactosidase from the GH 36 family, whose genes frequently occur in the genomes of marine bacteria, were characterized for the first time for recombinant α-galactosidase from the marine bacterium *Pseudoalteromonas* sp. KMM 701 (α-PsGal). As a result of our bioinformatic analysis of the amino acid sequence of the enzyme and homologous modeling of the 3D structure, presumably catalytic (D451 and D516) and substrate-binding (C494) residues—extremely important for the functioning of the enzyme—were identified. The predicted nucleophilic residue D451 and substrate-binding residue C494 were replaced with A451 and N494, respectively. Properties of the mutant D451A and C494N were investigated with comparison to wild α-PsGal. 

We showed that α-PsGal and its mutant C494N are cold-active enzymes characterized by their neutral pH-optima (6.5–8.0) and low thermostability of 20 to 30 °C among the known α-galactosidases. The wild enzyme exhibited about 30% activity at 5 °C. No data on temperature and pH effects on the activity were available in the literature for the prototype α-galactosidases from the mesophiles *L. acidophilus.* The α-galactosidases from different mesophilic lactobacilli showed an acidic optimum activity, in the pH range from 5.2 to 5.9, and maximum activity between higher temperatures of 38 to 42 °C [23] compared with α-PsGal. The optimal temperature for the activity of the AgaA enzyme from psychrophilic lactic acid bacterium *Carnobacterium piscicola* was 32 to 37 °C [24]. The optimum temperature of the enzyme from *Lactobacillus fermentum* was found to be 45 °C. The enzyme was inactivated at temperatures higher than 55 °C and stable in wide ranges of temperatures and pH [25,26]. As for thermophilic enzymes from bacteria-thermophiles and hyperthermophiles *Bifidobacterium adolescentis* DSM 20083, *B. stearothermophilus*, *Thermus brockianus* [27], *Thermus sp. T2* [28], *Thermoanaerobacterium polysaccharolyticum* [29], and *Thermotoga maritime,* their temperature optimums were 75 to 100 °C. The last enzyme was inactive at 30 °C [30].

Capability of catalyzing a transglycosylation reaction is an inherent property of all members of the retaining α-d-galactosidases of the GH 27 and GH 36 families [4]. The inverting α-d-galactosidases of the GH 110 family [31], as well as NAD^+^- and Mn^2+^-dependent α-d-galactosidases found in the family GH 4 [32], have lost their transglycosylation properties. To date, there is no information about the transglycosylation ability of α-d-galactosidases from the GH 97 and GH 57 families.

It is known that the first step in the catalytic reaction is cleavage of the glycosidic bond of the melibiose or pNP-α-Gal molecules, as well as the formation of the covalent galactosyl-enzyme intermediate. The molecules of Glc and pNP are leaving groups. In the second step, water or some carbohydrate molecules attack the covalent galactosyl-enzyme intermediate, and then hydrolysis or transglycosylation, respectively, can be observed. In the case where the substrate is an attacking molecule, we can observe an autocondensation reaction (Figure S5).

α-PsGal catalyzed synthesis with a total yield of transglycosylation products ranging from 6.0% to 12% (Table 6), similar to the known retaining bacterial galactosidases of the GH 36 family. It was difficult to identify the structures of the transglycosylation products without appropriate standards. However, the use of three methods (TLC, MALDI MS in conjunction with ESIMS/MS, and NMR) provided a suggestion of the relationships between the sugars’ molecular weights and the type of O-glycoside bonds in the synthesized oligosaccharides.

TLC is commonly used to analyze low-molecular-weight sugars and their derivatives that differ in the number of carbon atoms, configurations, and molecule sizes. If two carbohydrates have one of these three different characteristics, they can be separated [33]. Based on the results, we assumed that the spot with R_f_ of 0.35 corresponds to bihexoses, distinguishable from melibiose by the configuration of the stereocenter, but the spot with R_f_ of 0.26 corresponds to sugars distinct from melibiose by the degree of polymerization (Figure 7, lane 1). The ^1^H NMR signals of the anomeric atoms of the trisaccharides were not detected. However, the signal of [Hex_3_ + Na]^+^ at *m*/*z* 527 was observed in the MALDI MS (Figure S2a). The structure of the trisaccharide Hex-(1→4)-Hex-(1→6)-Hex was established by electrospray ionization tandem mass spectrometry as Gal-(1→4)-Gal-(1→6)-Glc (Figure S2b). The kinetic of accumulation and consumption of Gal-(1→4)-Gal-(1→6)-Glc was registered with the use of heavy-oxygen water (Figure 8b). In the NMR spectrum of the reaction mixture, we found the 1H signals of the anomer atoms (1→6)-α- and (1→4)-α-linked bigalactosides only. In this connection, we think that the spot with an R_f_ of 0.35 corresponds to two poorly shared (1→6)-α- and (1→4)-α-linked bigalactosides Gal-α(1→6)-Galα,β (**3**) and Gal-α(1→4)-Galα,β (**4**), respectively. This assumption was confirmed by EISMS-MS (Figure S2c).

Thus, when melibiose was used as the substrate, the enzyme synthesized the (1→6)-α-linked bigalactosides (Figure S2c), similar to all known melibiases of the GH 36 family [16,34,35,36,37,38,39,40,41,42] and to their closely-related GH 27 representatives [43,44,45,46,47,48,49,50,51,52]. Furthermore, α-PsGal formed the (1→4)-α-linked bigalactosides as described for mesophilic terrestrial α-d-galactosidases from *Bifidobacterium breve* 203 [35], *Lactobacillus reuteri* [16], and the acidic GH 27 family α-d-galactosidases (AgaBf3S) from the bacterium *Bacteroides fragilis*. The latter was able to transfer galactosyl residues from pNP-α-Gal in lactose Gal-β(1→4)-Glc with the efficiency and strict (1→4)-α-regioselectivity [52], whereas α-PsGal synthesized both (1→6)-α- and (1→4)-α-*O*-glycoside bonds in the bigalactosides from melibiose in the ratio of 9:1 at 20 °C and 5:1 at 8 °C. It is interesting to note that glucose, which is released from melibiose, did not participate in the transglycosylation reaction as an acceptor because its content in the mixtures was almost half of all products without any change in the course of the reaction (Table 3).

Similarly, we established the structure of the autocondensation products in the mixtures of α-PsGal and pNP-α-Gal (Figure 7, lanes 2 and 4, respectively). α-PsGal was able to produce novel compounds by catalyzing the autocondensation reaction of pNP-α-Gal. Both the substituted Gal_2_-pNP with (1→6)-α- and (1→3)-α-O-glycoside bonds and unsubstituted Gal_2_ with (1→6)-α- and (1→4)-α-*O*-glycoside bonds were found in the reaction mixture. The ratio of (1→6)-α-:(1→3)-α-linked Gal_2_-pNP was 7:1, but the ratio for unsubstituted (1→6)-α-:(1→4)-α-linked bigalactosides was 3:1 at 20 °C. The ratio of (1→6)-α-:(1→4)-α-linked bigalactosides reached up to 2:1 at 8 °C (Table 6).

The transglycosylation properties are well-studied for the highly thermoresistant GH 36 α-d-galactosidase from the hyperthermophilic bacterium *Thermotoga maritima* (TmGal36A). This enzyme catalyzes an autocondensation reaction with pNP-α-Gal as a substrate, forming substituted (1→2)-α-, (1→3)-α- and (1→6)-α-linked Gal_2_-pNP [22]. In total, the wild TmGal36A can produce up to 5.5% transglycosylation products. The mechanism of the hydrolysis and synthesis in TmGal36A is not favorable for the formation and breaking of the (1→4)-α-O-glycosidic linkage [22], unlike α-d-galactosidases from human intestine [34,35,36,37] and α-PsGal from marine bacterium.

The replacement of the predictive nucleophilic residue D451 to A451 in the active center led to complete loss of the ability of α-PGal to catalyze the hydrolysis. For unknown reasons, the rescue strategy, with an addition of the external nucleophilic sodium azide, proved to be ineffective in this case. Molar concentrations of sodium azide or sodium formate were unable to restore or increase the activity of the mutant D425G of α-d-galactosidase from archaeon *Sulfolobus solfataricus* [53]. Sodium azide did not inhibit any activity of the wild enzyme α-PsGal [10], but it did not restore the activity in its mutant D451A. Galactosyl-β-azide was not found both either of the reaction products of mutant D451A and pNP-α-Gal substrate, as occurred in the experiment with TmGal36A [54]. The D327G mutant of TmGal36A lost hydrolytic properties but retained glycosynthase properties and became an effective α-galactosynthase, which could produce various galactosylated disaccharides from galactosyl-β-azide as a donor and pNP-α(β)-galactosides as acceptors [55]. 

The mutation C494N changed the specificity for α-PsGal in the synthesis of O-glycoside bonds. Under the action of the C494N mutant on pNP-α-Gal, the yield of pNP-Gal-α-(1→6)-Gal (**6**) decreased, whereas pNP-Gal-(1→3)-α-Gal was not observed. In addition, the content of Gal-(1→4)-α-Gal (**4**) increased two-fold (Table 6). In the literature, it has been reported that the substitution of some bulk residues in the active site of α-d-galactosidase Aga A from *Bacillus stearothermophilus* KVE39 resulted in a 4.5-fold increase in the yield of substituted (1→3)-α-linked compared with pNP-Gal-α-(1→6)-Gal [37]. A number of single and double substitutions of protruded residues in the active site of α-d-galactosidase from *Bifidobacterium adolescentis* DSM 20083 led to an increase in the yield of the total transglycosylation products but they did not change the regioselectivity of the reaction [22,38].

## 4. Materials and Methods

### 4.1. Materials

The 4-nitrophenyl-α-d-galactopyranoside (pNP-α-Gal), melibiose (Gal-α-(1→6)-Glc), galactose (Gal), glucose (Glc), Bovine serum albumin (BSA), NaN_3_, and 2,5-dihydroxybenzoic acid were purchased from Sigma Chemical Company (St. Louis, MO, USA). Encyclo DNA-polymerase and enterokinase were purchased from Evrogen (Moscow, Russian Federation). Sodium phosphates, one- and two-substituted, were purchased from PanReac AppliChem GmbH (Darmstadt, Germany). IMAC Ni^2+^ Sepharose, Q-Sepharose, Mono-Q, and Superdex-200 PG were purchased from GE Healthcare (Uppsala, Sweden). Heavy-oxygen water was purchased from Component Reactive (Moscow, Russia).

### 4.2. Homology Model of α-PsGal 3D Structure

The target-template alignment customization of the modeling process and 3D model building of α-PsGalA (GenBank: ABF72189.2) were carried out using the Molecular Operating Environment version 2018.01 [14] package (Chemical Computing Group ULC: 1010 Sherbrooke St. West, Suite #910, Montreal, QC, Canada, H3A 2R7, 2018) using the forcefield Amber12: EHT. The α-d-galactosidase from *Lactobacillus acidophilus* NCFM (PDB code: 2XN2) with a high-resolution crystal structure was used as a template. The evaluation of structural parameters, contact structure analysis, physicochemical properties, molecular docking, and visualization of the results were carried out with the Ligand interaction and Dock modules in the MOE 2018.01 program (Chemical Computing Group ULC: 1010 Sherbrooke St. West, Suite #910, Montreal, QC, Canada, H3A 2R7, 2018). The results were obtained using the equipment of Shared Resource Center Far Eastern Computing Resource of Institute of Automation and Control Processes Far Eastern Branch of the Russian Academy of Sciences (IACP FEB RAS) [56] 

### 4.3. Production of Recombinant Enzymes

The recombinant wild α-d-galactosidase α-PsGal was produced as described earlier [13]. The D451A and C494N mutants were produced by polymerase chain reaction (PCR)-mediated site-directed mutagenesis using the full-length wild gene of α-PsGal. The mutations were inserted in the sequences of synthetic oligonucleotides for each DNA chain of the wild gene:

(1) D451A dir 5′-TTAAGTACATTAAATGGG**C**TATGAACCGCGA-3′ D451A rev 5′-GTTAATATCGCGGTTCATA**G**CCCATTTAATG-3′
(2) C494N dir 5′-AGGGCTTGAAATAGAAAGC**AA**TTCGTCAGGTGG-3′ C494N rev 5′-ACGTGCACCACCTGACGAA**TT**GCTTTCTATTTC-3′

The plasmid DNA pET40 containing an insertion of the α-d-galactosidase gene of the marine bacterium *Pseudoalteromonas* sp. KMM 701 (α-PsGal) or its D451A and C494N mutants were transformed in the *E. coli* strain Rosetta (DE3). Heterological expression was carried out at optimal conditions into *E. coli*, as described previously [57]. Purification of the recombinant α-PaGal and its D451A and C494N mutant forms were performed according to the procedures described previously [13].

### 4.4. Enzyme and Protein Essays

To determine the activity, 0.02 mL of an enzyme solution were mixed with 0.38 mL of the pNP-α-Gal solution (1 mg/mL in 0.05 M sodium phosphate buffer, pH 7.0). The reaction mixture was incubated at 20 °C for 10 min. The reaction was stopped by addition of 0.6 mL of 1 M Na_2_CO_3_. One unit of activity (U) was determined as the amount of enzyme that releases 1 μmol of pNP per 1 min at 20 °C in 0.05 M sodium phosphate buffer at pH 7.0. The amount of released pNP was determined spectrophotometrically (ε_400_ = 18300 M^−1^ cm^−1^). The specific activity was calculated as U/mg of protein. Protein concentration was determined by the Bradford method and calibrated with BSA as a standard [58].

#### 4.4.1. Circular Dichroism Spectra

The CD spectra were recorded in the ultraviolet (UV) region of 190 to 250 nm with Chirascan plus CD spectrometers (Applied Photophysics Ltd., Leatherhead, UK), equipped with an optional Peltier temperature controller for rapid and precise temperature control of the sample cell (Quantum North West, 22910 E Appleway Avenue, Suite 4 Liberty Lake, WA, USA), in 0.01 M sodium phosphate buffer (pH 7.0) and 20 °C. The average molecular weight of the amino acid residue for calculation of molar ellipticity [Ѳ] (degree cm^2^ dmol^−1^) was assumed to be 112 Da. The secondary structure elements were calculated by the Provencher–Glöcker method CONTIN/LL modified by Sreerama N. of the CDPro software package, 2000 (Colorado State University, Fort Collins, Colorado, USA, http://lamar.colostate.edu/sreeram/CDPro) [59,60]

#### 4.4.2. UV Absorption Spectra

Absorption spectra of proteins were recorded with a UV-Visible spectrophotometer UV-1601 PC (Shimadzu, Kyoto, Japan) in quartz cells with an optical path length of 1 cm, 0.1 cm, and 0.01 cm in the range of 190 to 400 nm. The molar extinction coefficient of enzyme ε_280_ = 100,770 M^−1^ cm^−1^ was calculated from the content of aromatic amino acids using the ExPASy server [61].

### 4.5. Effect of pH and Temperature

The pH optimums of purified enzymes were determined with pNP-α-Gal as the substrate in the pH range of 5.2 to 6.5 using 0.1 M sodium citrate buffer, in the pH range of 6.2 to 8.0 using 0.1 M sodium phosphate buffer, and in the pH range of 7.8 to 9.0 with 0.1 M Tris-HCl buffer. The temperature optimums for the purified enzymes were determined at pH 7.0 in the temperature range of 5 to 40 °C. The temperature stabilities of the enzymes were investigated after incubation for 60 min at 10 to 40 °C.

### 4.6. Determination of Kinetic Parameters

All kinetic studies were performed in 0.1 M sodium phosphate buffer, pH 7.0, at 20 °C. The Michaelis–Menthen constants, *K*_m_ and *V*_max_, were determined from the coefficients of linear regression of the Lineweaver–Burk plot. The substrate concentrations (mM) were 3.24, 2.59, 1.62, 1.29, 0.81, 0.65, 0.40, and 0.32 for wild α-PsGal and 2.50, 2.0, 1.25, 1.0, 0.62, 0.50, 0.31, and 0.25 for the C494N mutant.

### 4.7. Transglycosylation 

#### 4.7.1. Acceptor Specificity of Transglycosylation

For preliminary determination of acceptor specificity of transglycosylation, the synthesis reactions were performed at 20 °C for 24 h in a mixture (10 μL) containing 0.01 U of an enzyme, 10 mM of pNP-α-Gal or melibiose as the substrate, and 20 mM of glucose, galactose, fructose, fucose, or xylose as acceptor in 0.05 M sodium phosphate buffer (pH 7.0). The reaction was stopped by heating at 100 °C for 5 min and the reaction mixture was centrifuged at 14,000 rpm. Sugars were analyzed by TLC, mass spectrometry, and NMR spectroscopy methods.

#### 4.7.2. Transglycosylation Using Heavy-Oxygen Water (H_2_^18^O)

An experiment using mass spectrometry and heavy-oxygen water was prepared similarly as described in Section 4.7.1. above; but the concentrations were significantly lowered. Seven identical reaction mixtures were created. Each mixture contained 1.4 mg melibiose, 10 μL enzyme (1 U), and 70 μL H_2_O^18^ (0.02 M sodium phosphate buffer, 0.05 M NaCl, pH 7.0). The mixtures were incubated for 1, 2, 4, 6, 8, 12, and 24 h. Each reaction mixture was dissolved in 1 mL methanol and introduced into the mass spectrometer. For ESIMS and ESIMS-MS experiments, direct injection was performed using a syringe pump (KD Scientific, Hollison City, MA, USA) at a flow rate 5 µL/min. For LC-ESIMS experiments, samples were further diluted 10 times in methanol.

#### 4.7.3. Identification of Transglycosylation Products

The recombinant α-PsGal or D451A and C494N mutants, in an aqueous solution of 0.05 M sodium phosphate buffer (pH 7.0), were added to the preweighed dry samples of substrates melibiose or pNP-α-Gal or their mixture and were incubated for a certain time (Table 3) at 20 °C or 8 °C. The standard units of activity (U) or milligrams enzyme added (mg), the incubation time (τ) and reaction temperature are shown in Table 2. The reaction was stopped by heating at 100 °C for 5 min. The samples were centrifuged to remove the denatured protein and dried on Refrigerated CentriVap Concentrater (Labconco, Kansas City, MO, USA). The qualitative composition of the hydrolysis and transglycosylation products were analyzed by TLC and MALDI-MS without their separation from the reaction mixtures. Identification of oligosaccharides in the mixture and their output was performed via NMR spectroscopy.

#### 4.7.4. Thin-Layer Chromatography Analysis

Mono- and oligosaccharide composition of the hydrolysis and transglycosylation products were analyzed on silica gel TLC plates on aluminum foil (Sigma-Aldrich, St. Louis, MO, USA) with a 254 nm fluorescent indicator. The pore diameter was 60 A. R_f_ was calculated for every stain.

Weighed freeze-dried reaction product mixtures were placed in 0.5 mL Eppendorf, dissolved in distilled water to a concentration of 5 mg/mL, and centrifuged at 10,000 rpm to remove the denatured protein. A small spot of the analyzed mixture and standard compounds were applied at the start line of the TLC plate and chromatographed over 30 minutes in a sealed chamber Latch-Lid ChromatoTank (General Glass Blowing Co. Inc., Richmond, CA, USA), containing 100 mL of the mobile phase butanol/acetic acid/water (3:1:1; *v*/*v*/*v*). For visualization of stains, the plate was treated three times with 5% sulfuric acid solution, drying by warm air after each spraying.

#### 4.7.5. Mass Spectrometry Analysis

The molecular weights of the oligosaccharide ions were recorded as sodium adducts [M + Na]^+^ using MALDI time-of-flight mass spectrometer, Ultra Flex III (Bruker BioSpin GmbH, Rheinstetten/Karlsruhe, Germany) equipped with a smartbeam laser (355 nm, Bruker Daltonik GmbH, Bremen, Germany) in reflector mode at an accelerating voltage of 21 kV, using the saturated solution (acetonitrile-water, 1:1) of 2,5-dihydroxybenzoynoic acid as a matrix.

The composition of the oligosaccharide mixture after enzymatic transformation was performed using an Ultimate 3000 rapid separation liquid chromatography (RSLC) nano system (Dionex, Thermo Fisher Scientific, Waltham City, MA, USA) connected to a Bruker Impact II quadrupole time-of-flight (Q-TOF) mass spectrometer (Bruker Daltonics, Bremen, Germany). An Acclaim (Thermo Fisher Scientific, Waltham City, MA, USA) PepMap RSLC column (75 µm × 150 mm, C18, 2 μm, 100 A) was used for chromatographic separation. The mobile phases were 0.1% formic acid in H_2_O (eluent A) and 0.1% formic acid in acetonitrile (eluent B). The gradient program was: isocratic at 1% of eluent B from start to 5 min, from 1% to 10% eluent B from 5 to 10 min, from 10% to 95% eluent B from 10 to 11 min, and isocratic at 95% of eluent B to 15 min. After returning to the initial conditions, equilibration was achieved after 10 min. Chromatographic separation was performed at a 0.4 µL/min flow rate at 40 °C. Injection volume was 0.2 μL. The mass spectrometry detection was performed using CaptiveSpray (Bruker Daltonics, Bremen, Germany) ionization source at a capillary voltage of 1.3 kV. Collision induced dissociation (CID)-produced ion mass spectra were recorded in auto-MS/MS mode with collision energy 43 eV. The precursor ions were isolated with an isolation width of 1 mass unit.

The mass spectrometer was calibrated using the ESI-L Low Concentration Tuning Mix (Agilent Technologies, Santa Clara, CA, USA). The instrument was operated using the OTOFControl software (version 4.0, Bruker Daltonics, Bremen, Germany) and data were analyzed using Data Analysis software (version 4.3, Bruker Daltonics, Bremen, Germany).

#### 4.7.6. NMR Spectroscopy Analysis

The structure of disaccharides was characterized by NMR spectroscopy. Signals in the NMR spectra of sugars were assigned by two-dimensional correlated spectroscopy (H,H-COSY) and two-dimensional heteronuclear multiple bond correlation spectroscopy (HSQC) experiments. One-dimensional ^1^H-NMR and ^13^C-NMR, and two-dimensional H,H-COSY and HSQC spectra were recorded with a Bruker Avance III 500 HD (Bruker BioSpin GmbH, Rheinstetten/Karlsruhe, Germany) spectrometer in D_2_O at 50 °C with acetone as internal standard (δ = 31.45 and 2.20 ppm for ^13^C NMR and ^1^H NMR spectra, respectively). ^1^H NMR anomer signals of α-pNP-galactopyranose, β-galactopyranose, melibiose, and bigalactosides, as well as proton signals of the free 4-nitrophenol ring were analyzed and integrated by the standard software TopSpin 3.2.

The depth of the pNP-α-Gal conversion (H_pNPαGal_, %) was calculated by

H_pNPαGal_ = {I_Gal_/(I_pNPαGal_ + I_Gal2pNP_ + I_Gal2_)} × 100 (1)

where I_Gal_ is integrated intensities of all 1H signals of liberated Galα,β, I_pNPαGal_ is the integrated intensity of 1H proton signals in substrate pNP-α-Gal, and I_Gal2pNP_ and I_Gal2_ are the integrated intensity of 1H proton signals in transglycosylation products.

The depth of melibiose conversion (H_Mel_, %) was calculated by

H_Mel_ = (I_Gal_/I_Mel_) × 100 (2)

where I_Mel_ is the integrated intensities of signals 1H of initial mixture of melibiose.

Yield of oligosaccharides in the total reaction mixture (Y, %) was calculated by

Y = {I_product_ / (I_Gal_ + I_Gl__c_ + I_Gal2_ + I_Gal2pNP_)} × 100 (3)

where I_product_ is the integrated intensity of all 1H signals of a particular oligosaccharide.

## 5. Conclusions

The recombinant and mutated α-PsGal were shown to be expressed as soluble proteins with the use of pET-40 b (+)-based constructions, and were purified successfully with the His-tag approach. The wild α-PsGal from the cold-adapted marine bacterium *Pseudoalteromonas* sp. KMM 701 has the traits of a cold-active enzyme that catalyzes the hydrolysis and weak transglycosylation reactions. The combination of TLC, MALDI-MS, and NMR spectroscopy methods to analyze the reaction mixture sugars allowed us to define the regioselectivity of the transglycosylation reaction, to qualitatively and quantitatively identify the reaction products obtained under the action of the recombinant analogues of α-PsGal, and to evaluate the yield of these products. The yield of transglycosylation products ranged from 6 to 12%. α-PsGal has a narrow acceptor specificity but rather wide regioselectivity. D-glucose, D-fructose, L-fucose, and D-xylose are not acceptors in the transglycosylation reaction. Together with the major α(1→6)-links under experimental conditions, the enzyme produced minor (traces) α(1→4)- or -α(1→3)-links in bigalactosides at the saturating concentrations of melibiose and pNP-α-Gal, respectively. The point mutation D451A resulted in the completely loss of α-PsGal activity, indicating crucial significance of the residue A451 in the performance of the α-PsGal-mediated hydrolysis as well as transglycosylation. The C494N mutation slightly changed the structure, properties, and substrate specificity of the enzyme. Thus, *Pseudoalteromonas* KMM 701 α-d-galactosidase of the GH 36 family, which is important in biomedical technology, demonstrates weak glycosynthase properties in vitro.

## Figures and Tables

**Table 1:** Catalytic properties of wild α-PsGal and mutant C494N.

Enzyme	*K*_m_ (mM)	*V*_max_ (μmol min^−1^ ml^−1^)	*k*_cat_ (s^−1^)	*k*_cat_/*K*_m_ (mM^−1^ s^−1^)
Wild-type α-PsGal	0.40 ± 0.030	0.32 ± 0.003	324.4 ± 3.5	820
C494N mutant	0.30 ± 0.005	0.024 ± 0.0003	2.84 ± 0.02	3.86

Conditions of reactions: 0.05 M sodium phosphate, pH 7.0, 20 °C; concentration of α-PsGal: 34 μ/mL, mutant C494N: 22 μ/mL.

**Table 2:** Acceptor specificity of recombinant wild-type α-PsGal.

Substrate	Acceptor	New Products
pNP-α-Gal	d-Glucose	−
pNP-α-Gal	d-Fructose	−
pNP-α-Gal	d-Xylose	−
pNP-α-Gal	l-Fucose	−
pNP-α-Gal	d-Galactose	+
Gal-α(1→6)-Glcα,β	d-Xylose	−
Gal-α(1→6)-Glcα,β	d-Fructose	−
Gal-α(1→6)-Glcα,β	l-Fucose	−

(−) not detected; (+) detected.

**Table 3:** Composition of reaction mixtures for monitoring of transglycosylation with various forms of the recombinant α-galactosidase from the marine bacterium *Pseudoalteromonas* sp. KMM 701.

Enzyme	Substrate	Acceptor	Substrate (mol)	Acceptor (mol)	V (mL)	U or mg	t (°C)	τ (h)
Wild	Gal-α(1→6)-Glcα,β	-	0.11	0	0.1	2.2	20	48
Wild	Gal-α(1→6)-Glcα,β	-	0.31	0	0.3	4.5	8	7 days
Wild	pNP-α-Gal	-	0.05	0	0.2	1.0	20	48
Wild	pNP-α-Gal	-	0.07	0	0.2	0.015	8	7 days
Wild	Gal-α(1→6)-Glcα,β	pNP-α-Gal	0.06	0.13	0.3	2.6	20	48
C494N	pNP-α-Gal	-	0.07	0	0.3	0.012	8	7 days
D451A	Gal-α(1→6)-Glcα,β	NaN_3_	0.06	0.3	0.1	(0.008)	20	75
D451A	pNP-α-Gal	NaN_3_	0.07	0.3	0.3	(0.024)	20	75
D451A	pNP-α-Gal	-	0.07	0	0.2	(0.016)	20	75

**Table 4:** TLC and matrix-assisted laser desorption/ionization (MALDI)-mass spectrometry (MS) characteristics of reaction products of wild α-PsGal and its mutant C494N.

Enzyme	Substrate	Product Number	TLC (R_f_)	MALDI-MS (*m*/*z*)
Wild α-PsGal	Gal-α(1→6)-Glcα,β		0.39	365
		1	0.52	203
		2	0.55	203
		3	0.35	365
		4	0.35	365
		5	0.26	527
	pNP-α-Gal		0.83	324
		1	0.52	203
		6	0.61	486
		4	0.34	365
Mutant C494N	pNP-α-Gal		–	324
		1	–	203
		3	–	365
		6	–	486

**Table 5:** Anomer signals of nuclear magnetic resonance (NMR) spectra of substrates and products of hydrolysis and transglycosylation catalyzed by wild α-PsGal and its mutant C494.

Sugars		Product Number	δ_H_ (*J* in Hz)			δ_C_		
			A	B		A	B	
			αH1	αH1	βH1	αC1	αC1	βC1
Gal-α(1→6)-Glcα,β			5.00 (*3.7*)	5.24	4.67 (*7.8*)	98.2	92.2	96.1
A	B							
Galα,β		1		5.26 (*3.7*)	4.64 (*7.9*)		97.7	93.6
Glcα,β		2		5.23 (*3.7*)	4.58 (*7.8*)		97.6	93.6
Gal-α(1→6)-Galα,β		3	4.98 (*4.8*)	5.27	4.59 (*7.9*)	97.7		99.8
A	B							
Gal-α(1→4)-Glcα,β		4	5.22 (*3.2*)	N/D *		102.2	N/D	N/D
A	B							
Gal-(1→4)-Gal-(1→6)-Glc		5	undetected					
pNP-α-Gal				5.85 (*3.74*)			98.1	
Gal-α(1→6)-Gal-α-pNP		6	4.82 (*3.85*)	5.91 (*3.82*)		98.8	97.6	
A	B							
Gal-α(1→3)-Gal-α-pNP		7	5.87 (*3.6*)	N/D		N/D	N/D	
A	B							

* N/D = not defined.

**Table 6:** The yield and structure of the products of transglycosylation reactions catalyzed by the recombinant wild α-PsGal and its mutant C494N based on NMR data.

Enzyme	Substrate	T (°C)	Substrate Conversion (%)	Structure of the Hydrolysis and Transglycosylation Products		Yield of Products (%)
Wild	Gal-α(1→6)-Glcα,β	20	88.5	Gal	**1**	45.627.35.50.6
				Glc	**2**	
				Gal-α(1→6)-Galα,β	**3**	
				Gal-α(1→4)-Galα,β	**4**	
Wild	Gal-α(1→6)-Glcα,β	8	90.2	Gal	**1**	46.035.07.81.4
				Glc	**2**	
				Gal-α(1→6)-Galα,β	**3**	
				Gal-α(1→4)-Galα,β	**4**	
Wild	pNP-α-Gal	20	67.2	Gal	**1**	21.58.03.01.21.2
				Gal-α(1→6)-Gal-α-pNP	**6**	
				Gal-α(1→6)-Galα,β	**3**	
				Gal-α(1→4)-Galα,β	**4**	
				Gal-α(1→3)-Gal-α-pNP	**7**	
Wild	pNP-α-Gal	8	15.9	Gal	**1**	9.84.11.20.78<1
				Gal-α(1→6)-Gal-α-pNP	**6**	
				Gal-α(1→6)-Galα,β	**3**	
				Gal-α(1→4)-Galα,β	**4**	
				Gal-α(1→3)-Gal-α-pNP	**7**	
Wild	Gal-α(1→6)-Glcα,β + pNP-α-Gal	20	32.0	Gal	**1**	30.09.01.60.8
				Gal-α(1→6)-Gal-α-pNP	**6**	
				Gal-α(1→4)-Galα,β	**4**	
				Gal-α(1→6)-Galα,β	**3**	
C494N	pNP-α-Gal	8	19.0	Gal	**1**	5.02.82.01.2
				Gal-α(1→6)-Galα,β	**3**	
				Gal-α(1→6)-Gal-α-pNP	**6**	
				Gal-α(1→4)-Galα,β	**4**	

## Supplementary Materials

The following are available online at http://www.mdpi.com/1660-3397/16/10/349/s1, Figure S1: MALDI-MS of mixtures of reaction products with: (**a**) melibiose and pNP-α-Gal as donor and acceptor, respectively; (**b**) wild α-PsGal with pNP-α-Gal as substrate; (**c**) D451A mutant with pNP-α-Gal; (**d**) C494N mutant with pNP-α-Gal; and (**e**) MALDI-MS of cellobiose and melibiose as standards, Figure S2: Electrospray ionization mass (ESIMS-MS) spectra of the transglycosylation products catalyzed by wild α-PsGal with melibiose as substrate: (**a**) ESIMS-MS spectra; (**b**) fragmentation of *m*/*z* 527 ion; (**c**) fragmentation of *m*/*z* 365 ion, Figure S3: ESIMS-MS spectra of the transglycosylation products from pNP-α-Gal catalyzed by wild α-PsGal: (**a**) fragmentation of *m*/*z* 365 ion; (**b**) fragmentation of *m*/*z* 486 ion, Figure S4: ESIMS-MS spectra of the transglycosylation products catalyzed by mutant C494N: (**a**) melibiose as substrate; (**b**) fragmentation of *m*/*z* 365 ion; (**c**) pNP-α-Gal as substrate; (**d**) fragmentation of *m*/*z* 486 ion; (**e**) and fragmentation of *m*/*z* 347 ion. Figure S5: Schemes for presumable mechanism of hydrolysis and transglycosylation of substrates melibiose (**a**) and pNP-α-Gal (**b**) with recombinant α-PaGal, Table S1: Ligand Interactions Report.

## References

[1] Cantarel B.L., Coutinho P.M., Rancurel C., Bernard T., Lombard V., Henrissat B. (2009). The Carbohydrate-Active EnZymes database (CAZy): An expert resource for glycogenomics. Nucleic Acids Res..

[2] Henrissat B., Callebaut I., Fabrega S., Lehn P., Mornon J.P., Davies G. (1995). Conserved catalytic machinery and the prediction of a common fold for several families of glycosyl hydrolases. Proc. Natl. Acad. Sci. USA.

[3] Koshland D.E. (1953). Stereochemistry and the mechanism of enzymatic reactions. Biol. Rev. Camb. Phil. Soc..

[4] Bakunina I.Y., Balabanova L.A., Pennacchio A., Trincone A. (2016). Hooked on α-d-galactosidases: from biomedicine to enzymatic synthesis. Crit. Rev. Biotechnol..

[5] Ivanova E.P., Bakunina I.Y., Nedashkovskaya O.I., Gorshkova N.M., Mikhailov V.V., Elyakova L.A. (1998). Incidence of marine microorganisms producing β-N-acetylglucosaminidases, α-d-galactosidases and α-N-acetylgalactosaminidases. Rus. J. Mar. Biol..

[6] Bakunina I.Y., Nedashkovskaya O.I., Alekseeva S.A., Ivanova E.P., Romanenko L.A., Gorshkova N.M., Isakov V.V., Zvyagintseva T.N., Mikhailov V.V. (2002). Degradation of fucoidan by the marine proteobacterium *Pseudoalteromonas citrea*. Microbiology.

[7] Bakunina I.Y., Ivanova E.P., Nedashkovskaya O.I., Gorshkova N.M., Elyakova L.A., Mikhailov V.V. (1996). Search for α-d-galactosidase producers among marine bacteria of the genus *Alteromonas*. Prikl. Biokh. Mikrobiol..

[8] Bakunina I.Y., Nedashkovskaya O.I., Kim S.B., Zvyagintseva T.N., Mihailov V.V. (2012). Diversity of glycosidase activities in the bacteria of the phylum *Bacteroidetes* isolated from marine algae. Microbiology.

[9] Bakunina I.Y., Nedashkovskaya O.I., Balabanova L.A., Zvyagintseva T.N., Rasskasov V.V., Mikhailov V.V. (2013). Comparative analysis of glycoside hydrolases activities from phylogenetically diverse marine bacteria of the genus *Arenibacter*. Mar. Drugs.

[10] Bakunina I.Y., Sova V.V., Nedashkovskaya O.I., Kuhlmann R.A., Likhosherstov L.M., Martynova M.D., Mihailov V.V., Elyakova L.A. (1998). α-d-galactosidase of the marine bacterium *Pseudoalteromonas* sp. KMM 701. Biochemisrty.

[11] Balabanova L.A., Bakunina I.Y., Nedashkovskaya O.I., Makarenkova I.D., Zaporozhets T.S., Besednova N.N., Zvyagintseva T.N., Rasskazov V.A. (2010). Molecular characterization and therapeutic potential of a marine bacterium *Pseudoalteromonas* sp. KMM 701 α-d-galactosidase. Mar. Biotechnol..

[12] Terentieva N.A., Timchenko N.F., Balabanova L.A., Golotin V.A., Belik A.A., Bakunina I.Y., Didenko L.V., Rasskazov V.A. (2015). The influence of enzymes on the formation of bacterial biofilms. Health Med. Ecol. Sci..

[13] Bakunina I.Y., Balabanova L.A., Golotin V.A., Slepchenko L.V., Isakov V.V., Rasskazov V.A. (2014). Stereochemical course of hydrolytic reaction catalyzed by alpha-galactosidase from cold adapTable marine bacterium of genus *Pseudoalteromonas*. Front. Chem..

[14] (2018). Molecular Operating Environment (MOE).

[15] Fredslund F., Hachem M.A., Larsen R.J., Sørensen P.G., Coutinho P.M., Lo Leggio L., Svensson B. (2011). Crystal structure of α-galactosidase from *Lactobacillus acidophilus* NCFM: Insight into tetramer formation and substrate binding. J. Mol. Biol..

[16] Wang Y., Black B.A., Curtis J.M., Gänzle M.G. (2014). Characterization of α-galacto-oligosaccharides formed via heterologous expression of α-d-galactosidases from *Lactobacillus reuteri* in *Lactococcus lactis*. Appl. Microbiol. Biotechnol..

[17] Menshova R.V., Ermakova S.P., Anastyuk S.D., Isakov V.V., Dubrovskaya Y.V., Kusaykin M.I., Um B.H., Zvyagintseva T.N. (2014). Structure, enzymatic transformation and anticancer activity of branched high molecular weight laminaran from brown alga *Eisenia bicyclis*. Carbohydr. Polym..

[18] Domon B., Costello C.E. (1988). A Systematic nomenclature for carbohydrate fragmentations in FAB-MS/MS spectra of glycoconjugates. Glycoconj. J..

[19] Zaia J., Miller M.J.C., Seymour J.L., Costello C.E. (2007). The role of mobile protons in negative ion CID of oligosaccharides. J. Am. Soc. Mass Spectrom..

[20] Weignerova L., Hunkova Z., Kuzma M., Kren V. (2001). Enzymatic synthesis of three pNP-α-galactobiopyranosides: application of the library of fungal α-d-galactosidases. J. Mol. Catal. B: Enzym..

[21] Borisova A.S., Ivanen D.R., Bobrov K.S., Eneyskaya E.V., Rychkov G.N., Sandgren M., Kulminskaya A.A., Sinnott M.L., Shabalin K.A. (2015). α-Galactobiosyl units: Thermodynamics and kinetics of their formation by transglycosylations catalysed by the GH36 α-d-galactosidase from *Thermotoga maritima*. Carbohydr. Res..

[22] Bobrov K.S., Borisova A.S., Eneyskaya E.V., Ivanen D.R., Shabalin K.A., Kulminskaya A.A., Rychkov G.N. (2013). Improvement of efficiency of transglycosylation catalyzed by α-d-galactosidase from *Thermotoga maritima* by protein engineering. Biochemistry.

[23] Mitfal B.K., Shallenberger R.S., Stainkraus K.H. (1973). α-Galactosidase activity of lactobacilli. Appl. Microbiol..

[24] Coombs J., Brenchley J.E. (2001). Characterization of two new glycosyl hydrolases from the lactic acid bacterium *Carnobacterium piscicola* Strain BA. Appl. Environ. Microbiol..

[25] Garro M.S., Degiori G.S., Devaldez G.F., Oliver G. (1993). Characterization of α-galactosidase from *Lactobacillus fermentum*. J. Appl. Bacteriol..

[26] Carrera-Silva E.A., Silvestroni A., LeBlanc J.G., Piard J.C., de Giori G.S., Sesma F. (2006). A thermostable α-galactosidase from *Lactobacillus fermentum* CRL722: Genetic characterization and main properties. Curr. Microbiol..

[27] Fridjonsson O., Watzlawick H., Gehweiler A., Rohrhirsch T., Mattes R. (1999). Cloning of the gene encoding a novel thermostable α-galactosidase from *Thermus brockianus* ITI360. Appl. Environ. Microbiol..

[28] Ishiguro M., Kaneko S., Kuno A., Koyama Y., Yoshida S., Park G.G., Sakakibara Y., Kusakabe I., Kobayashi H. (2001). Purification and characterization of the recombinant *Thermus* sp.. strain T2 α-galactosidase expressed in Escherichia coli. Appl. Environ. Microbiol..

[29] King M.R., White B.A., Blaschek H.P., Chassy B.M., Mackie R.I., Cann I.K. (2002). Purification and characterization of a thermostable α-galactosidase from *Thermoanaerobacterium polysaccharolyticum*. J. Agric. Food Chem..

[30] Liebl W., Wagner B., Schellhase J. (1998). Properties of an α-galactosidase, and structure of its gene galA, within an α- and β-galactoside utilization gene cluster of the hyperthermophilic bacterium *Thermotoga maritime*. Syst. Appl. Microbiol..

[31] Liu Q.P., Yuan H., Bennett E.P., Levery S.B., Nudelman E., Spence J., Pietz G., Saunders K., White T., Olsson M.L. (2008). Identification of a GH110 subfamily of α-1,3-galactosidases, novel enzymes for removal of the a3Gal xenotransplantation antigen. J. Biol. Chem..

[32] Liu Q.P., Sulzenbacher G., Yuan H., Bennett E.P., Pietz G., Saunders K., Spence J., Nudelman E., Levery S.B., White T. (2007). Bacterial glycosidases for the production of universal red blood cells. Nat. Biotechnol..

[33] Robyt J.F., Wilson I.D., Cooke M., Poole C.F. (2000). Carbohydrates/Thin-Layer (Planar) Chromatography. Encyclopedia of separation science.

[34] Van Laere K.M.J., Hartemink R., Beldman G., Pitson S., Dijkema C., Schols H.A., Voragen A.G.J. (1999). Transglycosidase activity of *Bifidobacterium adolescentis* DSM 20083 α-d-galactosidase. Appl. Microbiol. Biotechnol..

[35] Zhao H., Lu L., Xiao M., Wang Q., Lu Y., Liu C., Wang P., Kumagai H., Yamamoto K. (2008). Cloning and characterization of a novel α-d-galactosidase from *Bifidobacterium breve* 203 capable of synthesizing Gal-α-1,4 linkage. FEMS Microbiol. Lett..

[36] Cervera-Tison M., Tailford L.E., Fuell C., Bruel L., Sulzenbacher G., Henrissat B., Berrin J.G., Fons M., Giardina T., Jugea N. (2012). Functional analysis of family GH-36 α-d-galactosidases from *Ruminococcus gnavus* E1: Insights into the metabolism of a plant oligosaccharide by a human gut symbiont. Appl. Environ. Microbiol..

[37] Dion M., Nisole A., Spangenberg P., Andreé C., Glottin-Fleury A., Mattes R., Tellier C., Rabiller C. (2001). Modulation of the regioselectivity of a *Bacillus* α-d-galactosidase by directed evolution. Glycoconj. J..

[38] Hinz S.W.A., Doeswijk-Voragen C.H.L., Schipperus R., Broek L.A.M., Vincken J.P., Voragen A.G.J. (2006). Increasing the transglycosylation activity of α-d-galactosidase from *Bifidobacterium adolescentis* DSM 20083 by site-directed mutagenesis. Biotechnol. Bioeng..

[39] Nakai H., Baumann M.J., Petersen B.O., Westphal Y., Hachem M.A., Dilokpimol A., Duus J.Ø., Schols H.A., Svensson B. (2010). *Aspergillus nidulans* α-d-galactosidase of glycoside hydrolase family 36 catalyses the formation of α-galacto-oligosaccharides by transglycosylation. FEBS J..

[40] Spangenberg P., Andre C., Dion M., Rabiller C., Mattes R. (2000). Potential of new sources of α-d-galactosidases for the synthesis of disaccharides. Carbohydr. Res..

[41] Schroder S., Kroger L., Mattes R., Thiem J. (2015). Transglycosylations employing recombinant α- and β-galactosidases and novel donor substrates. Carbohydr. Res..

[42] Zhou J., Lu Q., Zhang R., Wang Y., Wu Q., Li J., Tang X., Xu B., Ding J., Huang Z. (2016). Characterization of two glycoside hydrolase family 36 α-d-galactosidases: Novel transglycosylation activity, lead–zinc tolerance, alkaline and multiple pH optima, and low-temperature activity. Food Chem..

[43] Koizumi K., Tanimoto T., Okada Y., Hara K., Fujita K., Hashimoto H., Kitahata S. (1995). Isolation and characterization of novel heterogeneous branched cyclomalto-oligosaccharides (cyclodextrins) produced by transgalactosylation with α-d-galactosidase from coffee bean. Carbohydr. Res..

[44] Spangenberg P., Andre C., Langlois V., Dion M., Rabiller C. (2002). α-Galactosyl fluoride in transfer reactions mediated by the green coffee beans α-d-galactosidase in ice. Carbohydr. Res..

[45] Savel’ev A.N., Ibatyllin F.M., Eneyskaya E.V., Kachurin A.M., Neustroev K.N. (1996). Enzymatic properties of α-d-galactosidase from *Trichoderma reesei*. Carbohydr. Res..

[46] Eneyskaya E.V., Golubev A.M., Kachurin A.M., Savel'ev A.N., Neustroev K.N. (1998). Transglycosylation activity of α-d-galactosidase from *Trichoderma reesei.* An investigation of the active site. Carbohydr. Res..

[47] Shabalin K.A., Kulminskaya A.A., Savel’ev A.N., Shishlyannikov S.M., Neustroev K.N. (2002). Enzymatic properties of α-d-galactosidase from *Trichoderma reesei* in the hydrolysis of galactooligosaccharides. Enzyme Microb. Technol..

[48] Shivam K., Mishra S.K. (2010). Purification and characterization of a thermostable α-d-galactosidase with transglycosylation activity from *Aspergillus parasiticus* MTCC-2796. Process Biochem..

[49] Wang C., Wang H., Ma R., Shi P., Niu C., Luo H., Yang P., Yao B. (2016). Biochemical characterization of a novel thermophilic α-d-galactosidase from *Talaromyces leycettanus* JCM12802 with significant transglycosylation activity. J. Biosci. Bioeng..

[50] Kurakake M., Moriyama Y., Sunouchi R., Nakatani S. (2011). Enzymatic properties and transglycosylation of α-d-galactosidase from *Penicillium oxalicum* SO. Food Chem..

[51] Vic G., Scigelova M., Hastings J.J., Howarth O.W., Crout D.H.G. (1996). Glycosydase catalysed synthesis of oligosaccharides: trisaccharides with the α-d-Gal-(1→3)-d-Gal terminus responsible for the hyper acute rejection response in cross-species transplant rejection from pigs to man. Chem. Commun..

[52] Gong W., Xu L., Gu G., Lu L., Xiao M. (2016). Efficient and regioselective synthesis of globotriose by a novel α-d-galactosidase from *Bacteroides fragilis*. Appl. Microbiol. Biotechnol..

[53] Brouns J.J.S., Smits N., Wu H., Snijders A.P.L., Wright P.C., de Vos W.M., van der Oost J. (2006). Identification of a novel alpha-galactosidase from the hyperthermophilic archaeon *Sulfolobus solfataricus*. J. Bacteriol..

[54] Comfort D.A., Bobrov K.S., Ivanen D.R., Shabalin K.A., Harris J.M., Kulminskaya A.A., Brumer H., Kelly R.M. (2007). Biochemical analysis of *Thermotoga maritima* GH36 α-d-galactosidase (*Tm*GalA) confirms the mechanistic commonality of clan GH-D glycoside hydrolases. Biochemistry..

[55] Cobucci-Ponzano B., Zorzetti C., Strazzulli A., Carillo S., Bedini E., Corsaro M.M., Comfort D.A., Kelly R.M., Rossi M., Moracci M. (2011). A novel α-d-galactosynthase from *Thermotoga maritima* converts β-d-galactopyranosyl azide to α-galacto-oligosaccharides. Glycobiology.

[56] Shared Resource Center Far Eastern Computing Resource of Institute of Automation and Control Processes Far Eastern Branch of the Russian Academy of Sciences (IACP FEB RAS). https://cc.dvo.ru.

[57] Golotin V.A., Balabanova L.A., Noskova Y.A., Slepchenko L.V., Bakunina I.Y., Vorobieva N.S., Terentieva N.A., Rasskazov V.A. (2016). Optimization of cold-adapted α-d-galactosidase expression in *Escherichia coli*. Protein Expr. Purif..

[58] Bradford M.M. (1976). A rapid and sensitive method for the quantitation of microgram quantities of protein utilizing the principle of protein-dye binding. Anal. Biochem..

[59] Provencher S.W., Glockner J. (1981). Estimation of globular protein secondary structure from circular dichroism. Biochemistry.

[60] Sreerama N., Woody R.W. (2000). Estimation of protein secondary structure from circular dichroism spectra: comparison of CONTIN, SELCON, and CDSSTR methods with an expanded reference set. Anal. Biochem..

[61] ExPASy (Expert Protein Analysis System) Proteomics Server. http://cn.expasy.org/tools/protparam.html.

