# Supplementary material for: Characterization of Properties and Transglycosylation Abilities of Recombinant α-Galactosidase from Cold-Adapted Marine Bacterium Pseudoalteromonas KMM 701 and Its C494N and D451A Mutants
Source: Mar Drugs. 2018 Sep 24;16(10):349. doi: 10.3390/md16100349 (PMC6213131; doi:10.3390/md16100349)
Supplement: Supplementary file 1 [file marinedrugs-16-00349-s001.pdf]

Supplementary Information

Figure S1. MALDI Mass Spectra of reaction product mixtures:

(**a**) wild α-PsGal with melibiose and pNP-α-Gal

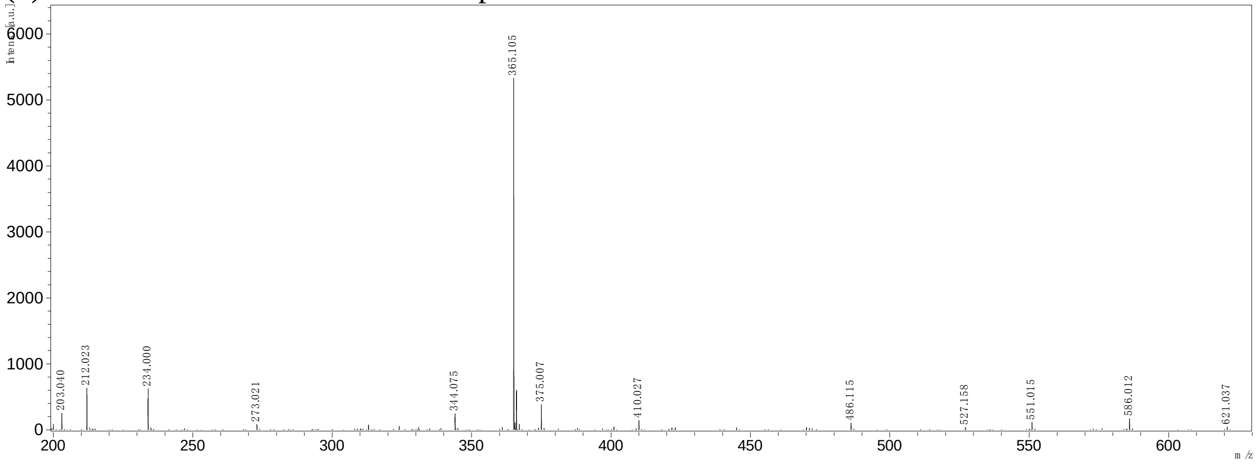

(**b**) wild α-PsGal with pNP-α-Gal

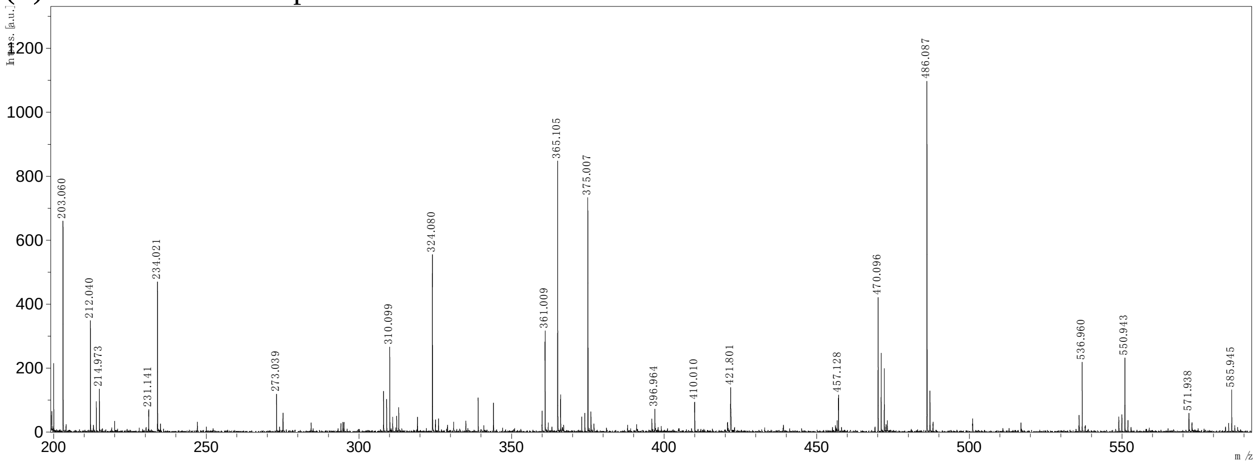

(**c**) D451A mutant with pNP-α-Gal

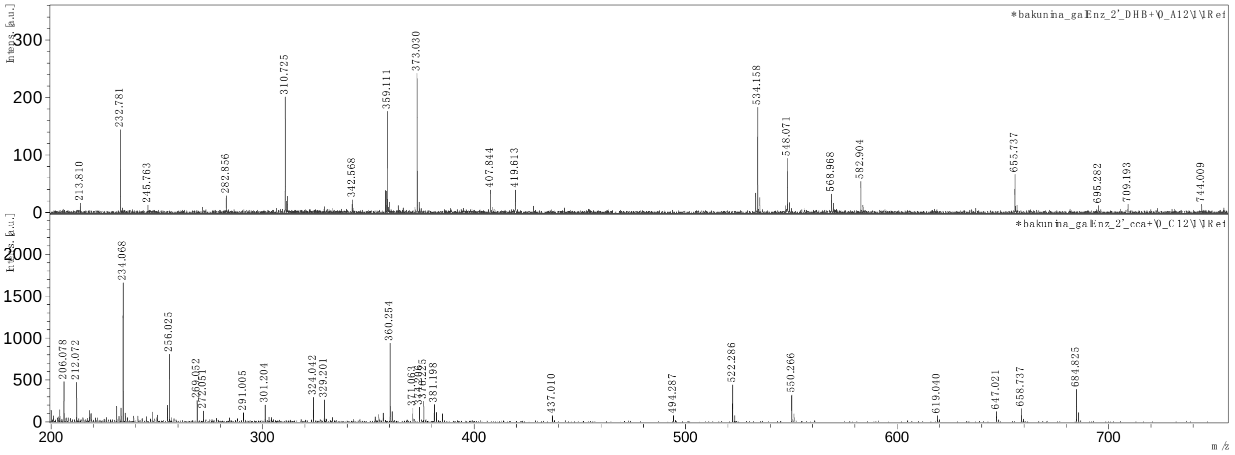

(**d**) C494N mutant with pNP-α-Gal

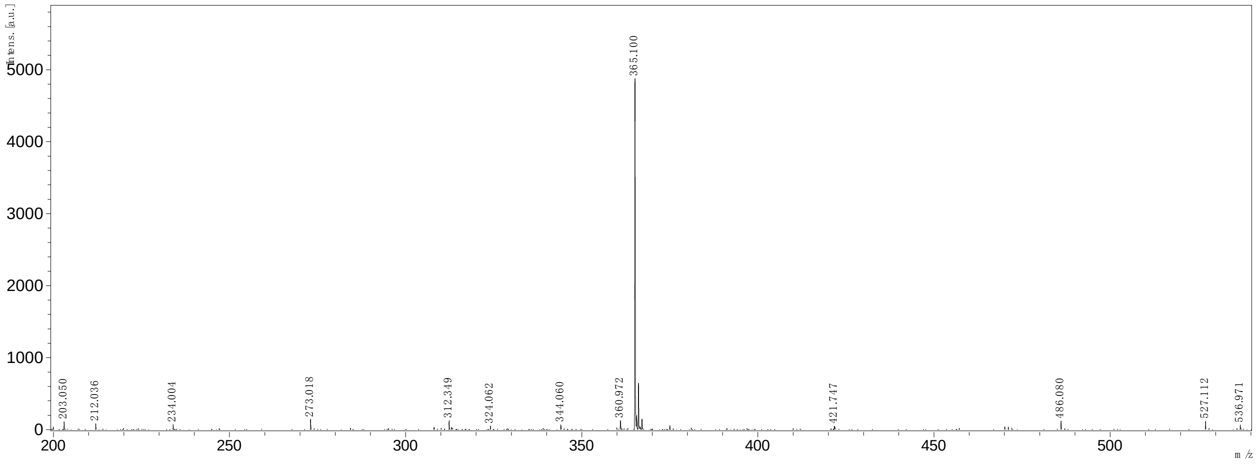

e) MALDI Mass Spectra of cellobiose and melibiose.

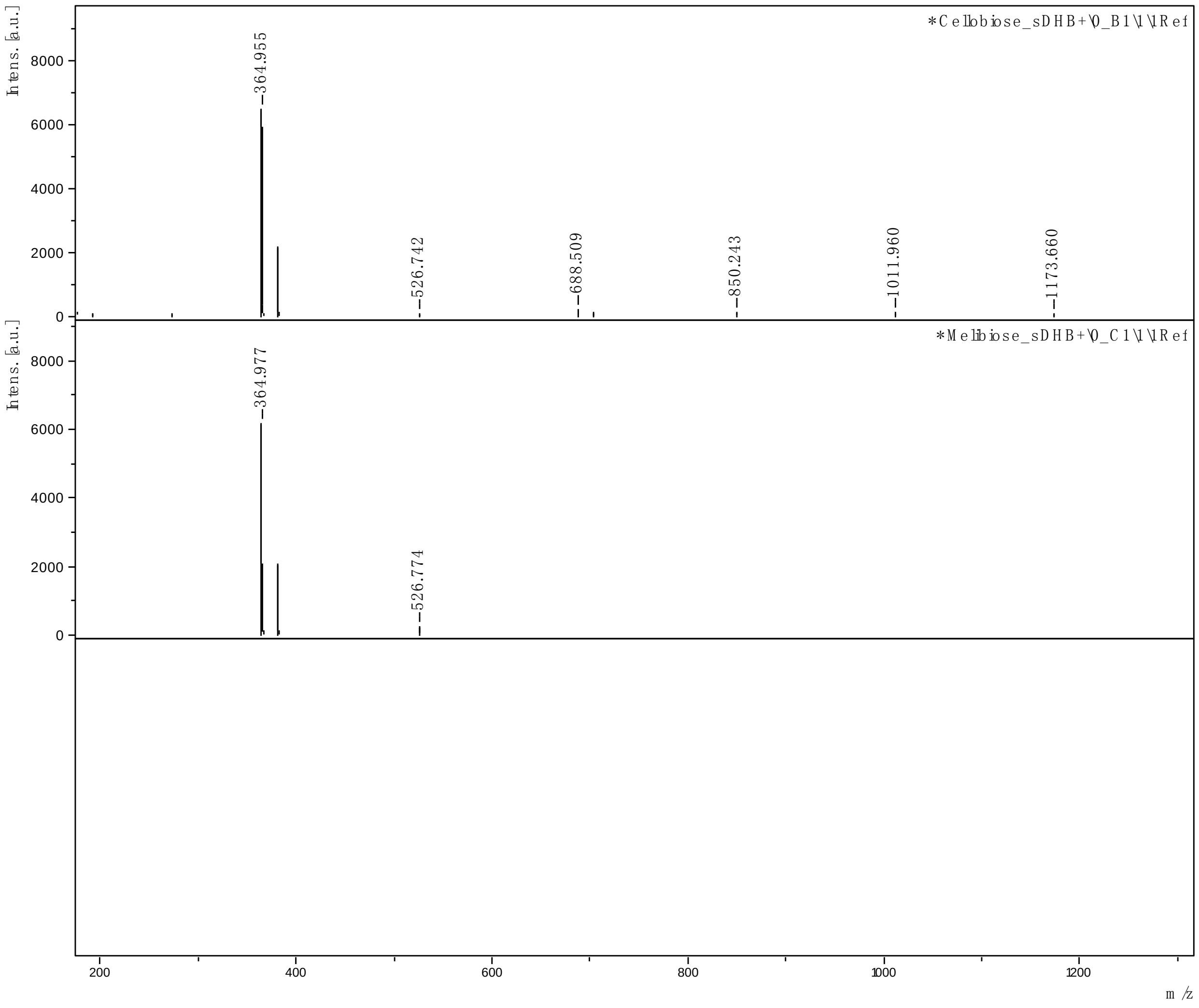

Figure S2. Electrospray ionization tandem mass spectrometry (ESIMS/MS) spectra of the transglycosylation products catalyzed by wild α-PsGal with melibiose as substrate.

a) (ESIMS/MS) spectra.

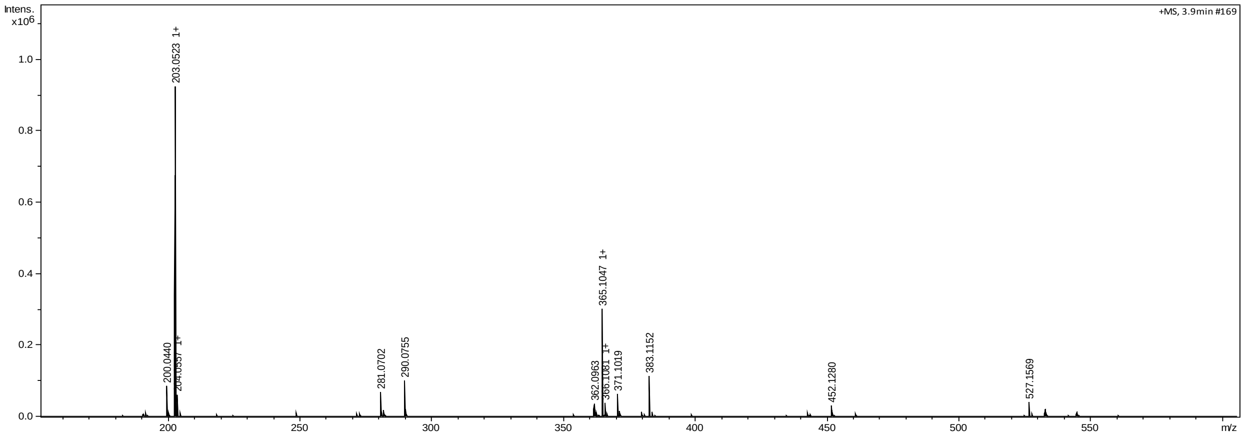

b) Fragmentation of 527 m/z ion

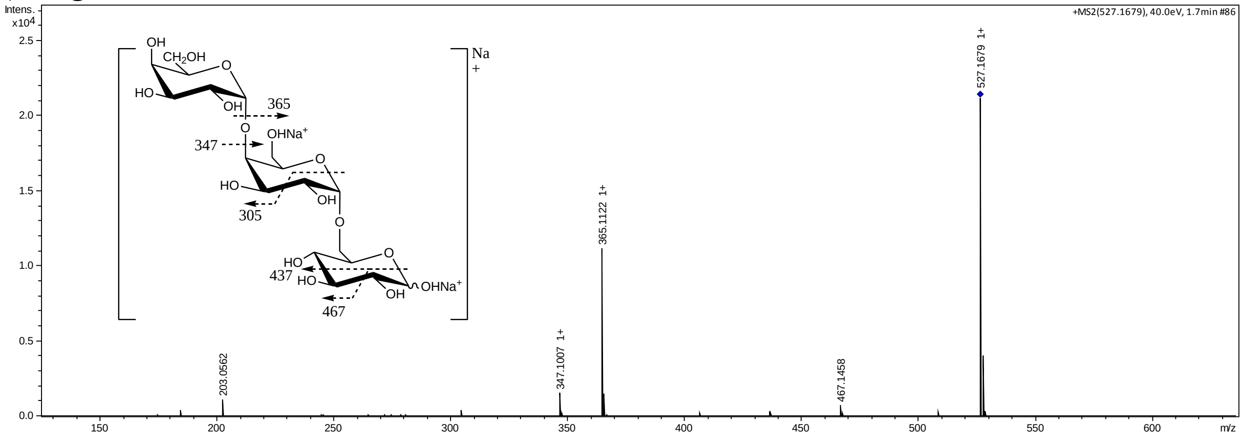

c) Fragmentation of 365 m/z ion

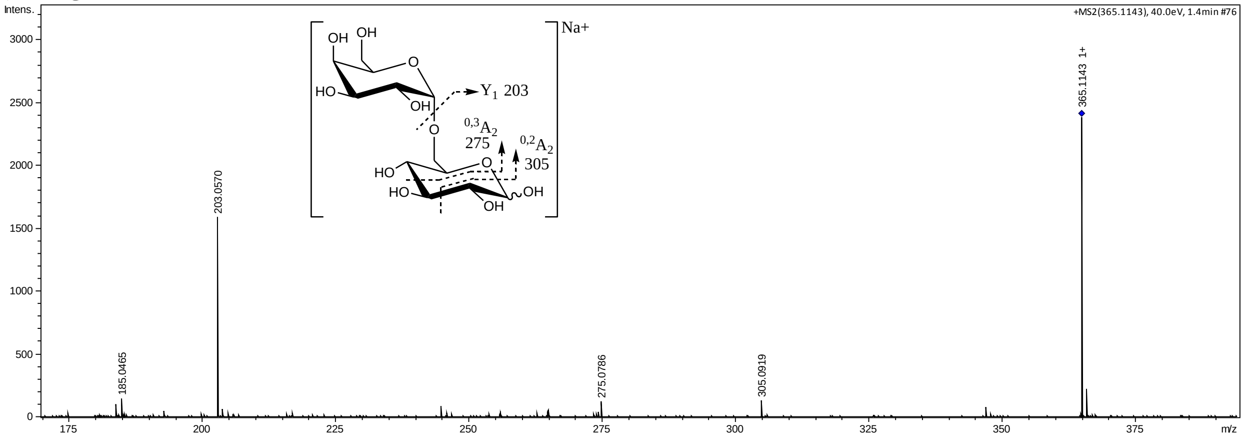

Figure S3. Electro spray ionization tandem mass spectrometry (ESIMS/MS) spectra of the transglycosylation products from pNP-galactoside catalyzed by wild α-PsGal.

a) Fragmentation of 365 m/z ion

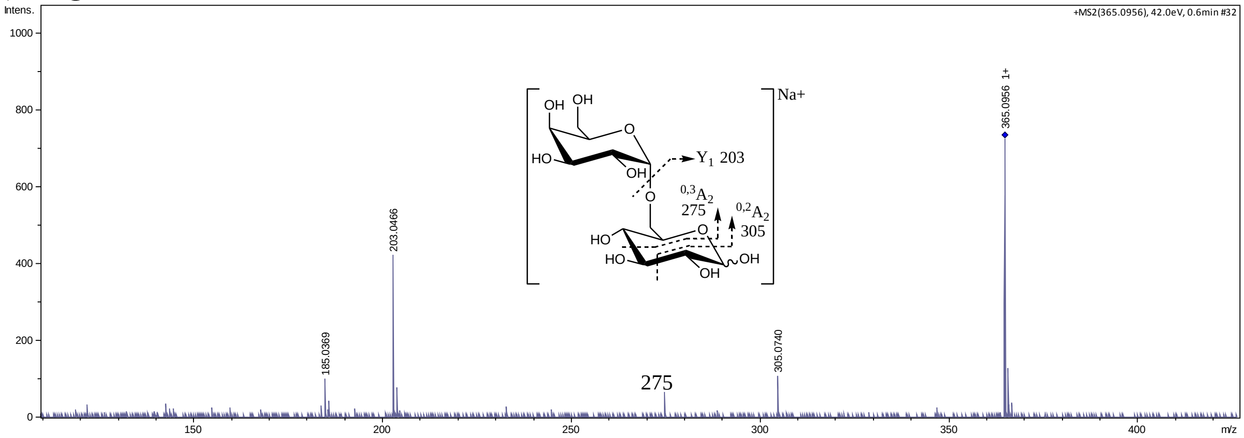

b) Fragmentation of 486 m/z ion

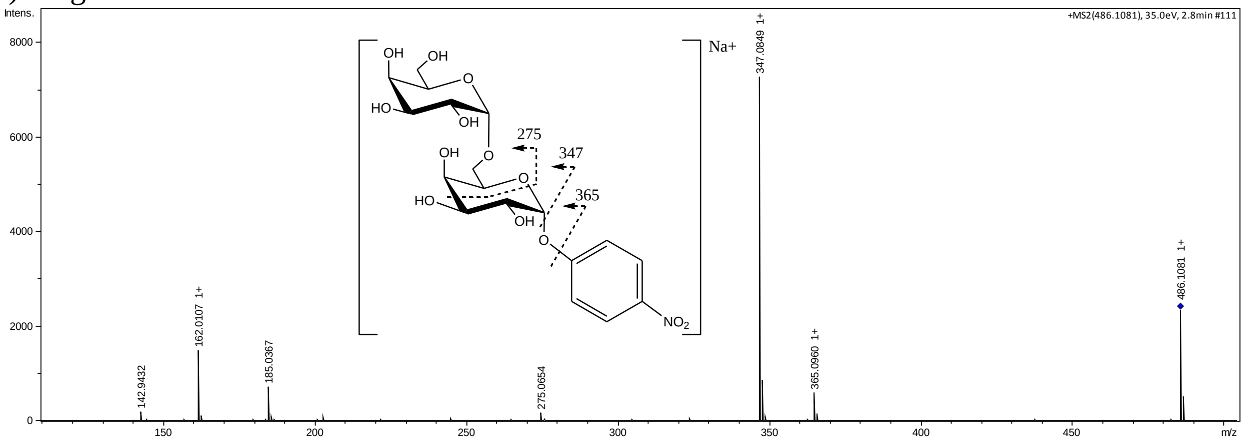

Figure S4. Electro spray ionization tandem mass spectrometry (ESIMS/MS) spectra of the transglycosylation products catalyzed by mutant C494N

a) Melibiose as substrate

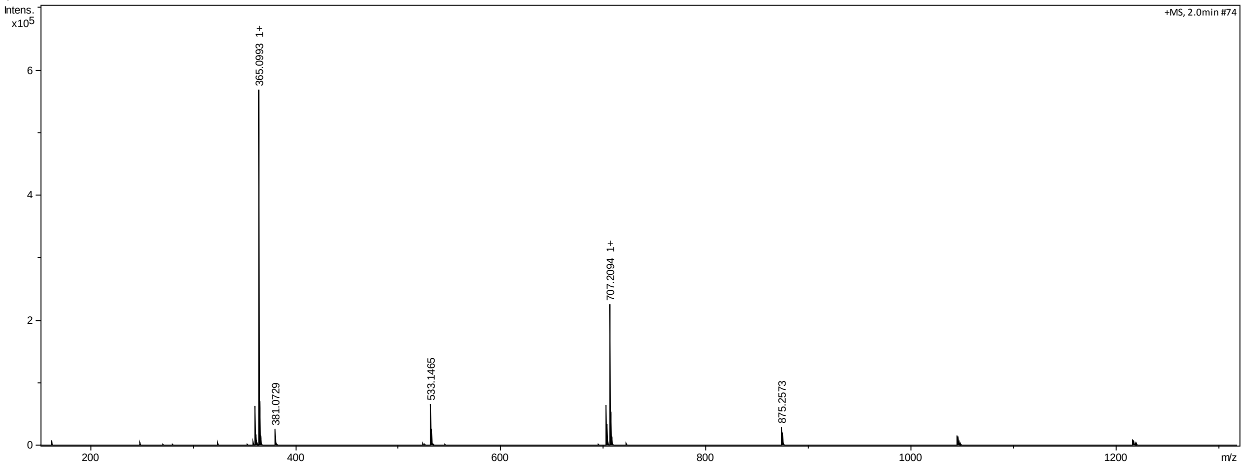

b) Fragmentation of 365 m/z ion

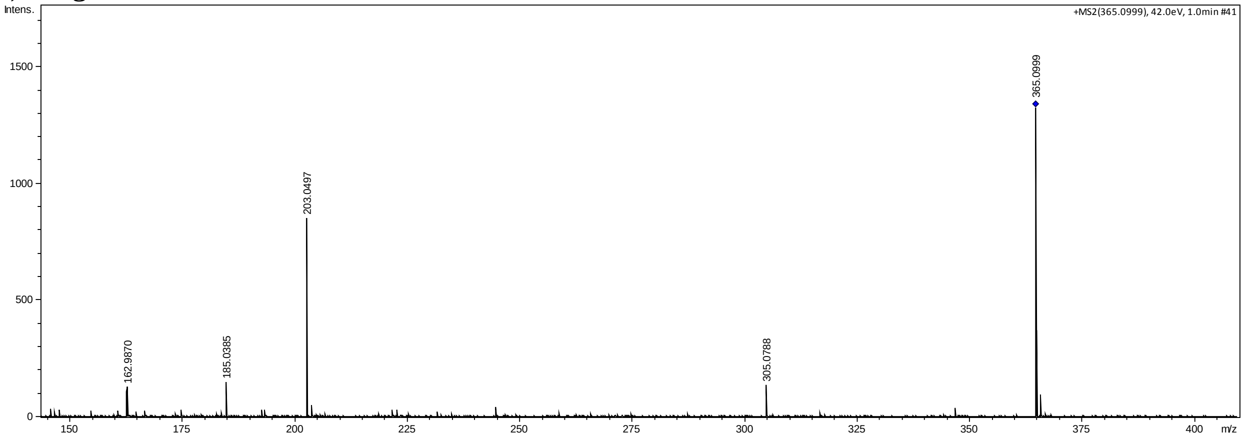

c) pNP-α-Gal as substrate

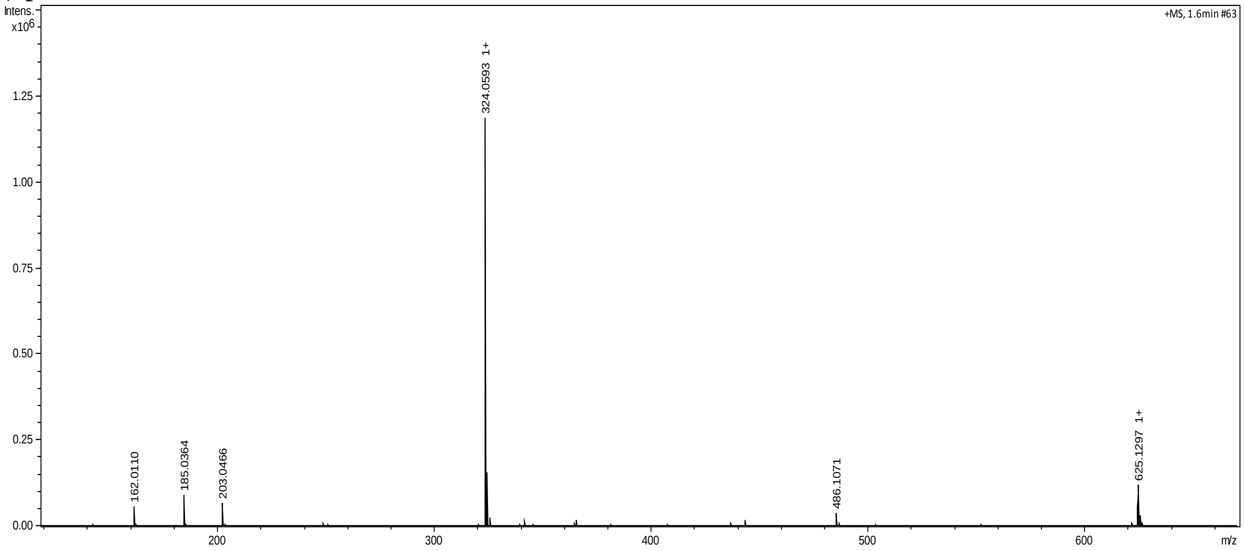

d) Fragmentation of 486 m/z ion

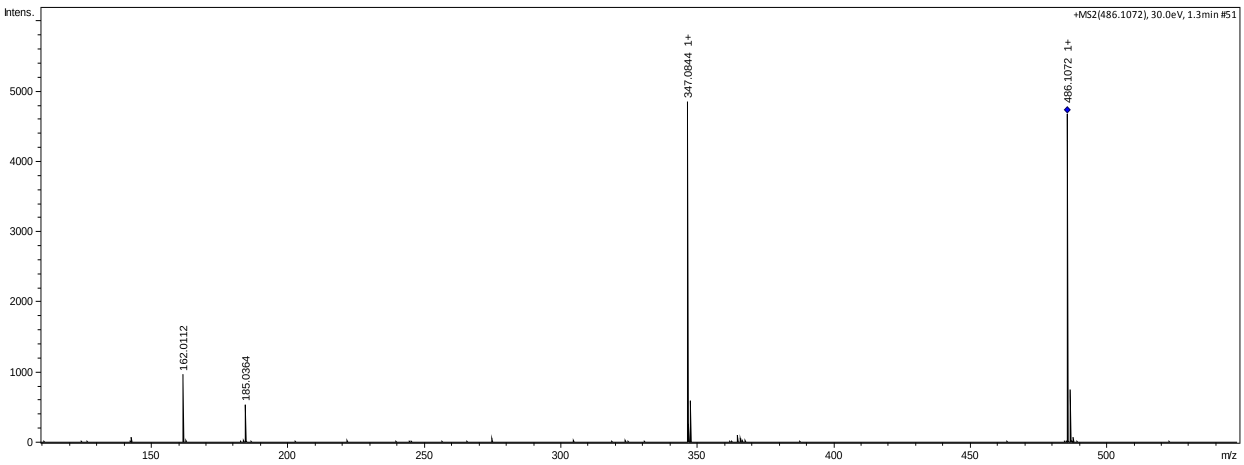

e) Fragmentation of 347 m/z ion

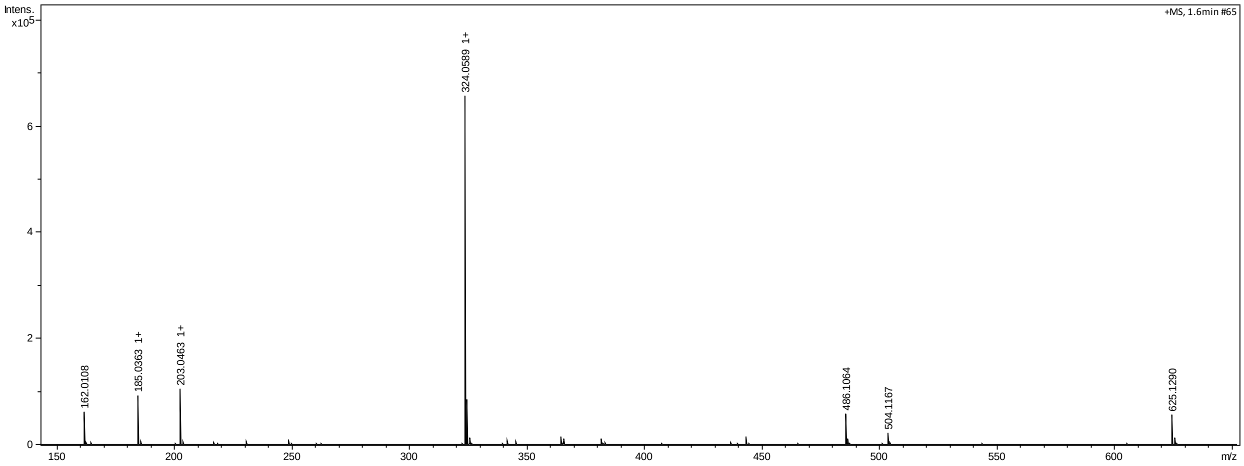

Figure S5. Schemes for presumable mechanism of hydrolysis and transglycosylation of substrates melibiose (a) and pNP-α-Gal (b) with recombinant α-PaGal. E – α-PsGal, Gal-(1→6)-α-Glc – melibiose, pNP-α-Gal – pNP-α-D-galactopyranoside, E : Gal-(1→6)-α-Glc and E : pNP-α-Gal – equilibrium complexes of α-PsGal and substrates, E-Gal – covalent enzyme-carbohydrate intermediate, (E : Gal : Gal)’ and (E : Gal : Gal)’’ – equilibrium double complexes for synthesis of major Gal-(1→6)-α-Gal and minor Gal-α-(1→4)-Gal, respectively, (E : Gal : pNP-α-Gal-)’ and (E : Gal : pNP-α-Gal)’’ equilibrium complexes for synthesis of major Gal-(1→6)-α-Gal-α-pNP and minor Gal-α-(1→3)-Gal-α-pNP, respectively.

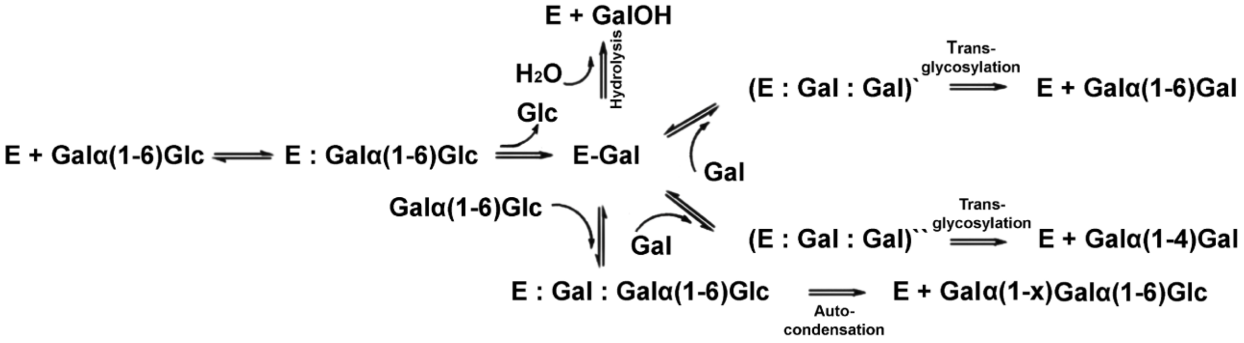

(a)

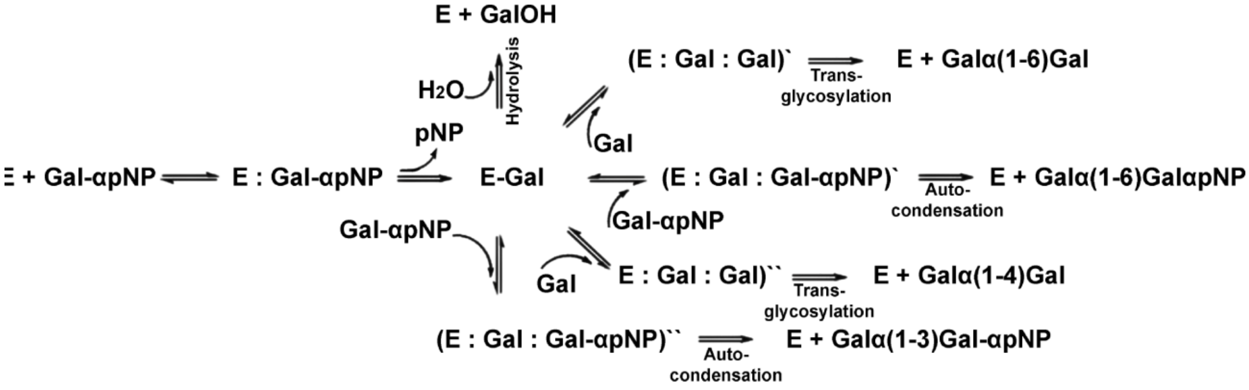

(b)

The first step of the catalytic reaction is a cleavage of the glycosidic bond of the melibiose or pNP-*α*-Gal molecules, as well as the formation of the covalent galactosyl-enzyme intermediate. The molecular of glucose (Glc) and pNP are leaving groups. On the second step water or some carbohydrate, molecules attack the covalent galactosyl-enzyme intermediate, and then hydrolysis or transglycosilation, respectively, can be observed. In the case when substrate is an attacking molecule, we observe the reaction of autocondensation.

The table S1 shows data for models of complexes obtained with the module Ligand Interaction of MOE program, as well as 2D-diagrams of the D-Gal binding sites in the wild α-PsGal and mutant C494N (Figure 7).

The 3D-structures of D-Gal complexes with the active centers of the α-PsGal and mutant C494N models used by MOE program module Ligand Interaction are shown in the picture for the reviewer. The figure shows the hydrogen bonds and the distances between the D-Gal atoms and the amino acid residues of the models.

Table S1 is supplemented with screenshots of the MOE program.

Table S1

Ligand Interactions Report

Tue Aug 07 11:50:44 2018 (MOE 2018.01)

wild PsGal/ D-Gal

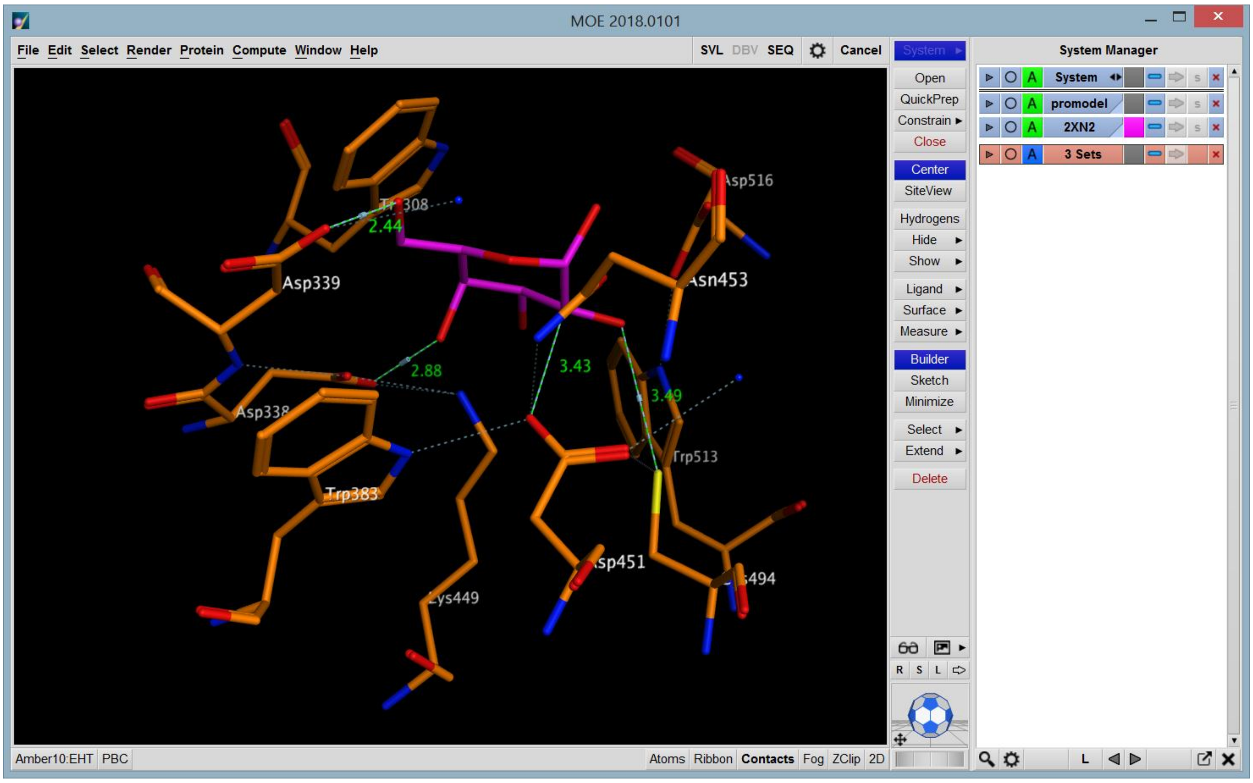

Ligand (kcal/mol)		Receptor			Interaction	Distance	E
C2	2	OD2	ASP	451	H-donor	3.43	-0.5
O2	8	SG	CYS	494	H-donor	3.49	-1.2
O4	10	OD1	ASP	338	H-donor	2.88	-2.2
O6	12	OD2	ASP	339	H-donor	2.44	-1.0
O2	8	N	GLY	497	H-acceptor	3.29	-0.9

mutant PsGal C494N / D-Gal

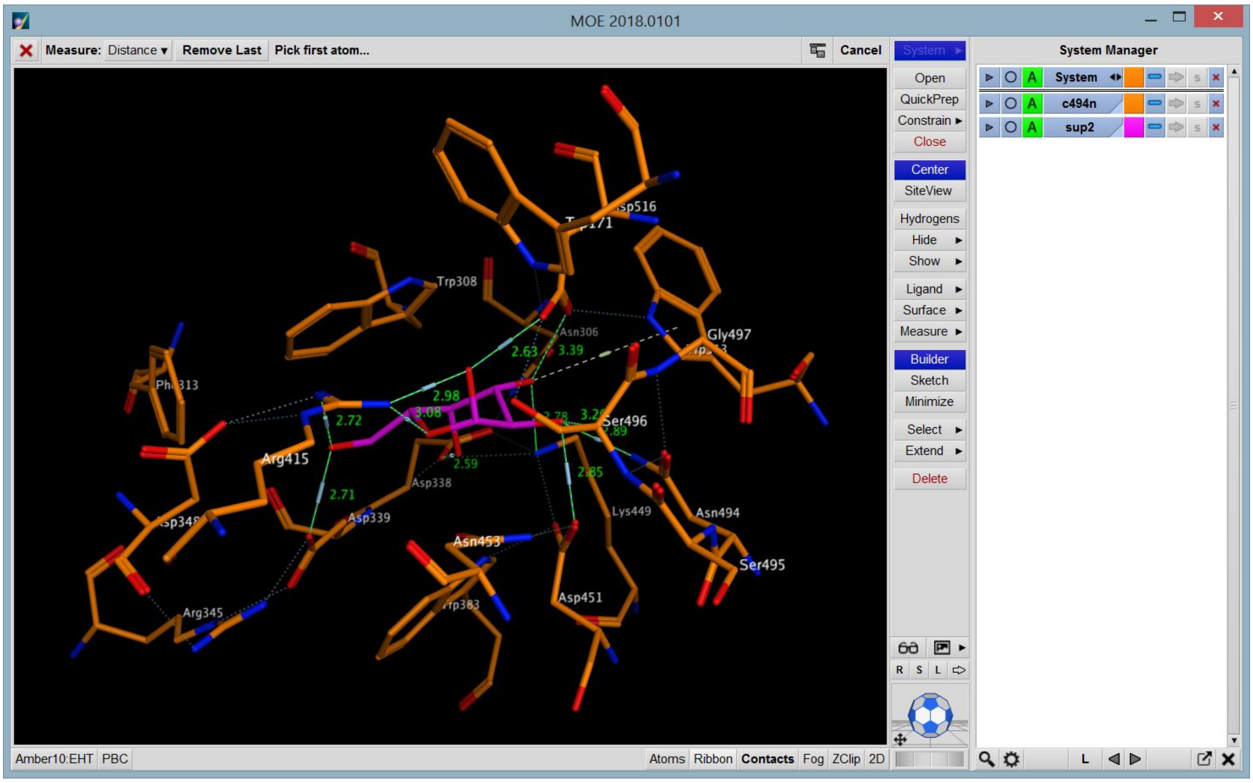

Ligand		Receptor			Interaction	Distance	E
(kcal/mol)							
O1	7	OD2	ASP	516	H-donor	2.63	-2.8
O2	8	OD1	ASP	451	H-donor	2.85	-3.8
O3	9	OD1	ASP	516	H-donor	3.39	-0.5
O4	10	OD2	ASP	338	H-donor	2.59	-3.5
O6	12	OD2	ASP	339	H-donor	2.71	-3.5
O1	7	NH1	ARG	415	H-acceptor	2.98	-3.2
O2	8	ND2	ASN	494	H-acceptor	2.89	-1.3
O2	8	CA	SER	496	H-acceptor	3.26	-0.5
O3	9	NZ	LYS	449	H-acceptor	2.78	-7.3
O5	11	NH1	ARG	415	H-acceptor	3.08	-1.1
O6	12	NH2	ARG	415	H-acceptor	2.72	-2.7
O3	9	5-ring	TRP	513	H-pi	4.52	-1.5
